# From Pixels to Stroma: AI-Driven Spatial Profiling of Cancer-Associated Fibroblasts on H&E and Its Implications for Immunotherapy

**DOI:** 10.3390/cancers18172802

**Published:** 2026-08-28

**Authors:** Dalani Tarun, Wong Kwun Hin Jerry, Jialin Wu, Xin Fang, Tiejun Feng, Fuda Xie, Muyang Huang, Yuanke Liang, Ka Fai To, Wei Kang, Haoyu Lin, Bonan Chen

**Affiliations:** 1 Department of Anatomical and Cellular Pathology, State Key Laboratory of Translational Oncology, Sir Y.K. Pao Cancer Center, The Chinese University of Hong Kong, Hong Kong SAR 999077, China; 2 Institute of Digestive Disease, State Key Laboratory of Digestive Disease, Li Ka Shing Institute of Health Science, The Chinese University of Hong Kong, Hong Kong SAR 999077, China; 3 CUHK-Shenzhen Research Institute, Shenzhen 518000, China; 4 School of Basic Medicine and Clinical Pharmacy, China Pharmaceutical University, Nanjing 211198, China; 5 Department of Thyroid and Breast Surgery, Clinical Research Center, The First Affiliated Hospital of Shantou University Medical College, Shantou 515000, China

**Keywords:** cancer-associated fibroblasts, computational pathology, artificial intelligence

## Abstract

Immune checkpoint blockade (ICB) has transformed cancer therapy, but clinical responses remain heterogeneous across tumor types and patient populations. Cancer-associated fibroblasts (CAFs) are key stromal components of the tumor microenvironment and can contribute to immunotherapy resistance through immune exclusion, extracellular matrix remodeling, chemokine signaling, and interactions with suppressive immune cells. Although CAF-directed strategies are under active investigation, their clinical translation is limited by marked CAF heterogeneity and the lack of scalable biomarkers for patient stratification. Computational pathology based on hematoxylin and eosin (H&E) whole-slide images (WSIs) provides a potential approach for extracting stromal and spatial features from routine histology, although digitized WSIs and the infrastructure required for large-scale AI analysis are not universally available. In this review, we synthesize current evidence on CAF classification, CAF-mediated immunotherapy resistance, H&E-based computational pathology, and emerging histology-based biomarker models. We further propose a conceptual roadmap for developing CAF-aware H&E spatial signatures with potential relevance to future immunotherapy stratification. Current evidence supports the biological rationale and computational feasibility of this approach, whereas its clinical utility remains to be established through rigorous external validation and prospective clinical evaluation.

## Simple Summary

Immune checkpoint inhibitors have improved cancer treatment, but many patients do not respond to these therapies. One important reason is the presence of cancer-associated fibroblasts (CAFs)—cells within the tumor that can block immune cells from reaching and attacking the cancer. While drugs targeting CAFs are being developed, their success depends on identifying which patients are most likely to benefit. This review explores whether artificial intelligence (AI) can analyze routine pathology slides to reveal CAF-related patterns that may predict immunotherapy response. We propose a research roadmap for developing such tools, which could eventually help guide treatment decisions alongside existing biomarkers. Although this approach is scientifically promising, it remains an area of active investigation and is not yet ready for routine clinical use.

## 1. Introduction

Various clinical trials have demonstrated that immune checkpoint blockade (ICB) on PD-1, PD-L1, and CTLA-4 has a strong impact on the treatment of various cancers. Notable examples include the Checkmate 067 trial for advanced melanoma [1], the KEYNOTE-024 trial for non-small-cell lung cancer (NSCLC) [2], the KEYNOTE-052 trial for urothelial cancer [3], the Checkmate 142 trial for colorectal carcinoma (CRC) [4], the IMbrave150 study for hepatocellular carcinoma (HCC) [5], and the ATTRACTION-2 study for gastric cancer (GC) [6]. Despite their results, the response rate of ICB is less than thirty percent in most cases. Moreover, these studies show a large variance in response rates. Part of it is contributed by the biomarkers used in assessing response, including PD-L1 immunohistochemistry, tumor mutational burden and microsatellite instability (MSI), or mismatch repair deficiency. This is an issue of stratification rather than drug development, which highlights the variable effectiveness of the same drug towards different tumor types. 

A potential source of the problem lies within cancer-associated fibroblasts (CAF), the predominant cell of the desmoplastic stroma. It has been documented as one of the key players in immune exclusion, as evidenced in a study by Mariathasan et al., which showed that TGF-β signaling between CAFs contributed to T cell exclusion from the urothelial tumor mass, which weakened the response to immunotherapy [7]. Similar phenomena were also reported from a study by Tauriello et al. on metastatic colorectal cancer specimens [8], and a pan-cancer analysis by Chakravarthy et al. also reported similar findings [9]. While targeting CAFs is a possible option to overcome immunotherapy resistance, their heterogeneity in functions is a barrier to precise targeted therapy, as highlighted in a review by Sahai et al., which revealed their variations from single-cell transcriptomics. Their review proposes the retention of CAFs with anti-tumorigenic functions as a focal point and the need for further understanding of CAFs to achieve this goal, such as a nomenclature system to identify CAFs and their functions [10].

Regarding CAF analysis, routine H&E slides are widely available in pathology practice, whereas digitized WSIs and the infrastructure required for large-scale artificial intelligence (AI) analysis are not universally available. Although H&E staining is relatively inexpensive compared with spatial omics, whole-slide scanning and digital pathology deployment remain resource-intensive. Nevertheless, computational pathology may enable the extraction of subtle stromal features from digitized H&E images and support scalable investigation of CAF-associated morphology. The review paper by Bera et al. summarizes the general framework for AI in digital pathology, where digitized WSIs are analyzed by either convolutional neural networks (CNNs), where the model learns task-relevant image features under a given supervision and aggregation strategy, or the hand-crafted approach, where pathologists manually define parameters for feature extraction [11]. Another review paper by Shmatko et al. noted that AI in digital pathology goes beyond morphological analysis and predicts useful clinical information, such as prognosis and treatment response, along with molecular information, such as genetic alterations and expressions [12].

Current H&E-based immunotherapy biomarkers are mostly tumor-centric or lymphocyte-centric, whereas CAF-mediated immune exclusion represents a missing stromal layer. Thus, in this review, we synthesize the current evidence and propose a conceptual translational roadmap for deriving CAF-associated spatial information from H&E WSIs and evaluating its potential value for future immunotherapy stratification. Alongside, we illustrate the heterogeneity of CAFs in the era of single-cell and spatial omics and their role in immunotherapy. Moreover, we explore the evolution of computational pathology on WSIs, from CNNs to foundation models, and predict CAF biology and immunotherapy markers from them. Lastly, we present future directions and possible challenges with computational pathology on CAFs.

## 2. Method

This article was designed as a narrative review with a structured literature search to integrate current evidence on cancer-associated fibroblast (CAF) heterogeneity, CAF-mediated immunotherapy resistance, and H&E-based computational pathology, with particular emphasis on their convergence in CAF-aware immunotherapy stratification. The aim was to provide a critical and translational synthesis rather than a systematic quantitative review. PubMed, Web of Science, and Scopus were searched from database inception to June 2026 using combinations of terms related to “cancer-associated fibroblasts”, “CAF heterogeneity”, “single-cell”, “spatial omics”, “immunotherapy”, “immune checkpoint blockade”, “H&E”, “whole-slide imaging”, “computational pathology”, “deep learning”, “multiple-instance learning”, and “foundation models”. Boolean operators were applied to maximize search sensitivity. To identify additional eligible studies, reference lists of relevant original articles and reviews were manually screened.

The literature was synthesized qualitatively because of substantial heterogeneity in study designs, experimental systems, clinical endpoints, and computational approaches. Mechanistic studies were emphasized for CAF–immune interactions, single-cell and spatial studies for CAF heterogeneity and organization, clinical studies for immunotherapy-related evidence, and original computational studies for H&E-based prediction. Divergent findings were retained and interpreted in the context of cancer type, CAF subtype, spatial setting, and study design.

The review was prepared with reference to SANRA principles, with emphasis on transparency of the literature search, balanced presentation of evidence, and appropriate interpretation of heterogeneous findings.

## 3. CAF Heterogeneity in the Era of Single-Cell and Spatial Omics

### 3.1. From the Activated Stroma to a Stratified Taxonomy

Historically, CAFs were identified using specific markers such as alpha-smooth muscle actin (α-SMA), fibroblast activation protein (FAP), and others. However, they fail to address the heterogeneity of CAFs [13]. A study by Chen et al. revealed four subtypes of CAFs. These include the progenitor CAF which serves as the initial developmental stage of CAFs, inflammatory CAFs (iCAF) which trigger immunological responses, myofibroblastic CAFs (myCAF) which induce contraction of the tumor mass, and matrix-producing CAFs (matCAF) which are involved in ECM synthesis [13]. A study by Ohlund et al. demonstrates the distinction between iCAFs and myCAFs, with the former having low α-SMA and high IL-6 expression, and vice versa for myCAFs [14]. Additionally, another subtype, called antigen-presenting CAFs (apCAF), was discovered by Elyada et al. and functions to stimulate CD4+ T cells [15]. Further subtypes, as discovered by Bartoschek et al., include angiogenesis-producing vascular CAFs (vCAF), with cycling CAFs as their proliferating segments, matrix-remodeling CAFs, and developmental CAFs, which contribute to tumorigenesis [16]. Functional classifications also exist, as evidenced by studies from Kieffer et al. and Costa et al., showing the CAF-S1 cluster with immunosuppressive effects [17,18]. Canonical markers of CAFs include α-SMA, FAP, podoplanin, S100A4, platelet-derived growth factor receptors alpha and beta, vimentin, LRRC15, CD146, Thy-1, periostin, and COL11A1 [19]. Due to the heterogeneity, no single marker is sufficient to define a CAF. Importantly, these CAF classifications should not be interpreted as fixed or mutually exclusive cell lineages. Cell lineage refers to developmental origin, whereas CAF phenotype, transcriptional state, spatial niche, and functional program describe distinct but potentially overlapping aspects of fibroblast identity. Accordingly, commonly used labels, such as myCAF, iCAF, apCAF, matCAF, LRRC15+ CAF, and CAF-S1 states, may capture different dimensions of CAF biology and are not necessarily directly equivalent across studies. Their interpretation therefore depends on tumor type, species, assay platform, clustering resolution, and functional context.

### 3.2. Pan-Cancer Fibroblast Atlases

Pan-cancer fibroblast atlases are useful tools for comprehending CAFs. For instance, a pan-cancer atlas by Galbo et al. showed that specific pan-CAF subtypes were resistant to anti-PD1 and anti-PD-L1 immunotherapy [20]. A single-cell analysis done by Luo et al. revealed the three-stage trajectory of CAF activation and revealed a type of CAF arising from the endothelial-to-mesenchymal transition that interacted with SPP1-positive tumor-associated macrophages to induce angiogenesis [21]. A multi-omic analysis performed on multiple tissues by Foster et al. identified three superclusters of CAFs, namely the quiescent steady-state-like CAFs, mechanoresponsive CAFs involved in mechanotransduction signaling, and immunomodulatory CAFs. These superclusters are conserved throughout multiple tissue types [22]. Another example of such conservation is a study by Jia et al., which unveiled that the biomarker COL11A1 is conserved throughout activated CAFs in epithelial malignancies [23]. The demonstration of the four CAF subtypes performed by Chen et al., which also revealed matCAF’s status as a poor prognostic factor and potential therapeutic target, is another illustration of the use of pan-cancer atlases [13]. Pan-cancer atlases can also reveal the spatial relations of different CAFs. This is demonstrated by a study from Cords et al., which not only revealed the types and distributions of CAFs on breast cancer samples, but also validated samples of NSCLC, which highlighted that an increase in myCAFs in the stroma reduces the proximity of immune cells to tumor cells, but an increase in iCAFs in the tumor compartment increases the above proximity [24,25].

### 3.3. Origin and Plasticity

CAFs originate from a variety of tissues. Such examples include resident fibroblasts from a study by Buechler et al. [26], mesothelial transition as evidenced by Huang et al. [27], mesenchymal stromal cells as illustrated by Burt et al. [28], pericytes as evidenced by Hosaka et al. [29], and adipocyte trans-differentiation as revealed by Miyazaki et al. [30]. A recurrent marker associated with a myofibroblastic CAF state is LRRC15. LRRC15+ CAFs exhibit substantial overlap with myofibroblastic and matrix-associated programs and have been associated with poor response to immunotherapy, as reported by Dominguez et al. and Krishnamurty et al. [31,32]. A crucial property of CAFs is their plasticity. A study by Biffi et al. demonstrated that the addition of TGF-β downregulates IL-1 receptors, preventing the IL-1-induced JAK/STAT pathway that forms iCAFs, and instead shifts the development towards myCAF in pancreatic stellate cells [33]. Moreover, a study by Crozier et al. showed that tumor cells can convert iCAFs to myCAFs through a DPP4 and YAP1 mechanism, which contributes to immunosuppression [34].

### 3.4. Spatial Niches and Cellular Neighborhoods

CAFs form spatial niches with other cells within a tumor that carries functional and prognostic implications. An important consideration is the impact it has on tumor immunity, which, based on Hegde and Chen, can be classified into inflamed tumors where the immune cells are in proximity to the tumor mass; immune-desert, where the tumor cells are surrounded by no immune cells; and immune-excluded, where immune cells are separated from the tumor mass by a stroma [35]. A study by Bell et al. revealed that iCAF predominance gave way to myCAF predominance as pancreatic intraepithelial neoplasia progressed to pancreatic ductal adenocarcinoma, with this transition being associated with an immune-desert phenotype [36]. Moreover, a study by Sathe et al. demonstrated that, in secondary hepatic colorectal cancer metastases, the spatial co-localization of CAFs and SPP1-positive tumor-associated macrophages was associated with an immune-excluded microenvironment [37]. Pantaleo et al. further described the spatial and temporal dynamics of CAF niches in breast cancer, showing that iCAF- and myCAF-enriched regions displayed distinct relationships with immune and tumor compartments over time [38]. Table 1 summarizes representative CAF states and immune associations across study contexts.

## 4. CAFs as Architects of Immunotherapy Resistance

### 4.1. T-Cell Exclusion as the Central Paradigm

**Figure 1:**
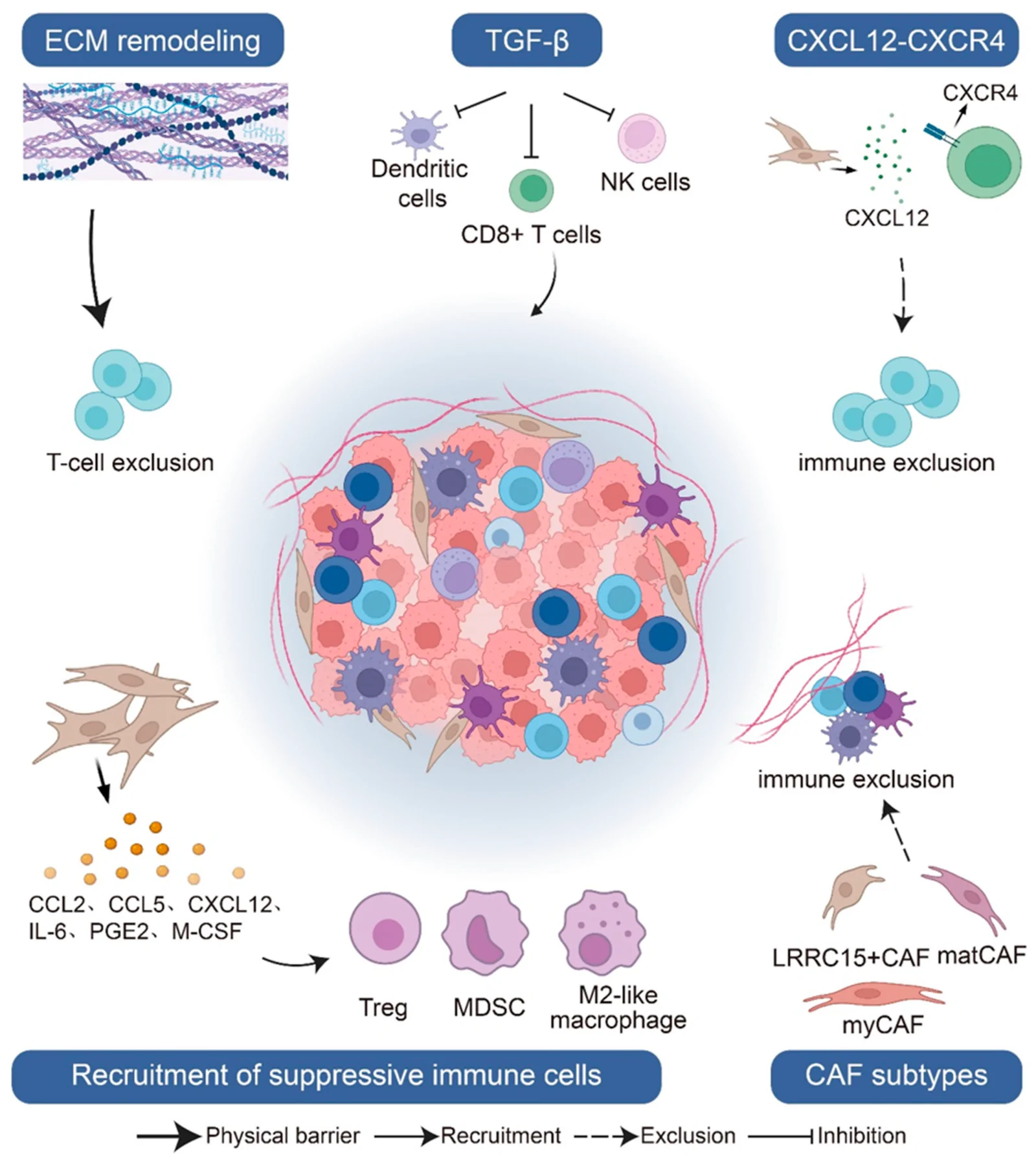
CAF-associated mechanisms of immune exclusion. CAFs can contribute to immune exclusion through context-dependent mechanisms, including ECM-mediated physical barriers, CAF-derived cytokine or chemokine-mediated recruitment of suppressive immune cells, TGF-β-associated inhibition of effector immune function, and CXCL12-CXCR4-associated exclusion. CXCL12 is shown as CAF-derived, whereas CXCR4 is indicated on responsive immune or tumor cells depending on context. Arrow styles distinguish recruitment, exclusion, inhibition, and physical-barrier mechanisms.

Collectively, these studies suggest that CAFs can contribute to immunotherapy resistance through multiple mechanisms, among which immune exclusion is one of the best-characterized. A study by Mariathasan et al. demonstrates immunosuppression mediated by TGF-β signaling between CAFs, and that adding TGF-β-blocking antibodies alongside anti-PD-L1 antibodies reduces intercellular signaling, thereby enhancing immunotherapy efficacy in urothelial carcinoma samples [7]. The same phenomenon is also observed in a study by Tauriello et al. on samples of CRC [8]. Additionally, a pan-cancer study by Chakravarthy et al. revealed the existence of tumor subgroups based on their response to anti-PD-L1 therapy: immune-hot with a lower CAF abundance and immune-cold with a higher CAF abundance [9]. There are four main mechanisms behind T-cell exclusion due to CAFs. Kuczek et al. revealed that collagen density reduces T-cell proliferation in breast cancer specimens [44]. Pruitt et al. revealed that the reduced parallelity of collagen fibers reduces T-cell mobility to the tumor cells in prostate cancer specimens [45]. Xiao et al. revealed that a denser stroma reduces T-cell penetration to the tumor mass [46]. And Feig et al. showed that CXCL12 from FAP-expressing CAFs contributes to T cell exclusion through CXCR4-dependent signaling in the tumor microenvironment [47]. Figure 1 summarizes the major mechanisms of immunosuppression by CAFs. These findings also suggest that PD-L1 immunohistochemistry alone may not fully capture the stromal mechanisms that influence immunotherapy response.

### 4.2. Subtype-Specific Contributions to Checkpoint Blockade Outcome

Besides the general mechanisms above, specific subtypes of CAFs have unique mechanisms that also confer immunosuppression. As aforementioned, LRRC15+ myCAFs confer immunosuppression via T-cell exclusion, which is evidenced by Dominguez et al. and Krishnamurty et al. [31,32]. Krishnamurty’s study also revealed that ablation of LRRC15+ myCAFs increases T cell activity against tumor cells [32]. From the study by Kieffer et al., myCAF-S1 (cluster 0) upregulates PD-L1 and CTLA4 in regulatory T cells, which then increases the levels of myCAF in cluster 3, which further increases the level of regulatory T cells and forms a positive feedback loop, thus increasing their immunosuppressive effects [17]. Moreover, from the study by Huang et al., apCAFs can induce the formation of regulatory T cells from naive helper T cells, and targeted therapy against mesothelin from mesothelial cells can prevent the formation of apCAFs to reduce regulatory T cell levels [27]. Lastly, a study by Yang et al. illustrates the secretion of IL-6 from iCAFs and the induction of myeloid-derived suppressor cell and regulatory T cell proliferation, the polarization of macrophages towards the M2 phenotype, and the reduction in cytotoxic T cell infiltration to tumor cells [33,40]. These studies illustrate subtype- and state-dependent mechanisms through which CAFs may contribute to immunosuppression and support further evaluation of CAF-specific features beyond global stromal measures. However, whether such features provide predictive value for ICB response requires validation in appropriately designed treated human cohorts.

### 4.3. Cross-Cancer Evidence

Cross-cancer studies have revealed context-dependent associations between CAF phenotypes and tumor immune states or immunotherapy outcomes. In NSCLC, Cords et al. showed that a high density of matrix CAFs was correlated with lower immune infiltration and poorer patient survival [24]. In HCC, POSTN-positive CAFs were associated with reduced T cell infiltration and poorer immunotherapy response, with mechanistic analyses further implicating IL-6/STAT3-mediated interactions with SPP1-positive macrophages [43]. In metastatic melanoma, higher SMA-positive CAF abundance was associated with poorer outcomes following anti-PD-1 therapy, although CAF parameters were not significantly associated with best overall response [48]. In *Helicobacter pylori* (*H. pylori*)-positive GC, THBS1-positive CAFs were spatially associated with regulatory T cells and an immune-excluded microenvironment [49]. A CAF-related gene signature in bladder cancer was associated with survival and immune features but was not directly evaluated in an ICB-treated cohort [50]. Conversely, HNCAF-0/3 populations in HNSCC were associated with favorable nivolumab response, with ex vivo experiments supporting reduced TGF-β-dependent CD8+ T cell dysfunction [51]. Taken together, these findings highlight the heterogeneous associations between CAF states, clinical outcomes, and immunotherapy response, while underscoring the need for treatment-specific validation before their predictive values can be established.

### 4.4. Stromal Therapeutics and the Stratification Bottleneck

To address the immunosuppressive effects of CAFs, stromal-directed therapies have been developed. Bispecific CAR-T cells, designed by Zhou et al., target FAP and GPC3, thereby engaging FAP-expressing CAFs and GPC3-expressing tumor cells in HCC [52]. Simlukafusp alfa (FAP-IL2v) is another drug studied by Waldhauer et al., in which the FAP component localizes IL-2 variant activity to FAP-positive stromal regions, and the IL-2 segment binds to T cells and NK cells to induce their proliferation [53]. Bintrafusp alfa is a bifunctional fusion protein that reduces local TGF-β by binding to tumor cells expressing PD-L1, anchoring the TGF-β-inhibiting domain to inhibit local TGF-β more effectively, as discovered by Lan et al. [54]. LRRC15-targeted radioligand or radio-immunotheranostic approaches, such as [177Lu]Lu-DUNP19, have been developed to enable non-invasive radiolocalization and local radiotherapy of LRRC15-positive CAF-rich lesions [55]. Moreover, NADPH oxidases 1 and 4 (NOX1/4) are targets for inhibition because they are selectively expressed in CAFs. The dual inhibition results in decreased TGF-β signaling mediated by NOX4, and inhibiting NOX1 prevents the compensatory increase in signaling, as evidenced by Amengual et al. [56]. Lastly, Andtbacka et al. investigated mavorixafor, an orally bioavailable CXCR4 antagonist that increased immune-cell infiltration and inflammatory activity in melanoma tumors [57]. In the CAF-related context, the more commonly discussed axis involves CAF-derived CXCL12 acting on CXCR4-positive tumor or immune cells, rather than mavorixafor principally binding CXCR4 on CAFs. While multiple drugs are available as potential targets, the main barrier to successful clinical application is patient selection for drug trials. To resolve this, artificial intelligence biomarkers derived from H&E can be considered to identify candidates for such therapies.

## 5. Computational Pathology on Hematoxylin and Eosin: From Convolutional Networks to Foundation Models

### 5.1. The Whole-Slide Image as a Digital Substrate

As previously mentioned, H&E slides are widespread in diagnostic pathology, but digitized WSIs suitable for AI analysis depend on scanning infrastructure, storage capacity, quality control, and deployment workflows. There are three important properties to consider before exploring their applications. Firstly, the gigapixel scale affects AI model interpretation. A study by Campanella et al. noted that the gigapixel scale of WSIs makes direct native-resolution analysis impractical because of computational and memory constraints. Their weakly supervised tile-based framework addressed this limitation by decomposing each slide into smaller image regions and aggregating slide-level evidence without abandoning whole-slide interpretation [58]. The next property is the variability of staining protocols and scanner platforms. A study by Lin et al. noted the ineffectiveness of normalizing staining protocols and AI training to reduce the impact of staining variability, and their study found that certain foundation AI models were durable against such variations [59]. The final property is image tessellation. A study by Ciga et al. highlighted the need for tessellation due to the large WSI size, but emphasized data curation bias at the patch level as an important limitation, in which the representation of malignant and benign tissue is mismatched towards malignant tissue due to pathologist annotations. Their study also proposed negative data sampling to reduce false-positive rates in tumor detection by clustering negative samples based on salient features, rather than randomly sampling negative regions, which may miss rare benign areas [60]. Methods of analysis are explored from the aforementioned framework; the predominant method in this review is via CNNs. Early examples of their use are demonstrated in a study by Coudray et al., which classified lung cancer samples as normal, adenocarcinoma, or squamous cell carcinoma, with diagnostic performance comparable to that of a pathologist [61]. Another example is a study by Kather et al., which demonstrated that AI models can predict MSI status from H&E images and may support screening or triage for cases requiring confirmatory immunohistochemistry or molecular testing [62].

### 5.2. Multiple-Instance Learning Frameworks

Classically, WSIs were interpreted using weakly supervised learning, in which a single label is assigned to a slide, and then tessellated. However, a major issue, highlighted in a study by Laleh et al., is that all tiles fall under the same label, regardless of their representability. One way to resolve this is to use multiple-instance learning models, in which representative tiles are weighted or aggregated for slide-level prediction [63]. Multiple MIL models have been developed. One example is the attention-guided multiple-instance learning (ABMIL) model developed by Yao et al.; image patches are assigned model-derived attention weights according to their contribution to the slide-level prediction [64]. Another example is the clustering-contained-attention multiple-instance learning model developed by Lu et al., which builds on the ABMIL model by the inclusion of instance-level clustering where tiles with the highest and lowest attention weights are used for instance-level clustering and representation learning on identifying benign vs. malignant tiles, and refining the feature space by clustering the two groups together to enhance diagnostic accuracy [65]. Furthermore, positional encoding-guided transformer-based MIL, developed by Shi et al., incorporated the spatial coordinates of tissue patches into their semantic features. It uses a position encoder to generate spatial-aware embeddings and a multi-head self-attention module to model the spatial and semantic relationships among patches [66]. Additionally, dual-stream MIL is developed by Li et al., which makes use of self-supervised contrastive learning to recognize normal tissue, followed by a dual-stream mechanism. The first stream identifies a critical instance, which the second stream then uses to assign attention scores to other tiles based on their resemblance to the critical instance for cancer diagnosis [67]. The final example is the additive multiple-instance learning (aMIL) developed by Markey et al., which employs a scoring system based on the presence of TGF-β CAFs. This enabled the visualization of regions contributing to predicted TGF-β-CAF signature scores based on the score distribution in the slide, where the top quartile of scores is labeled as “excitatory”, corresponding to high CAF density [68].

### 5.3. The Rise of Self-Supervised Learning and Pathology Foundation Models

Between 2023 and 2025, there has been a wave of novel self-supervised learning (SSL) and pathology foundation models, including self-supervised image encoders, whole-slide models, and vision–language systems, one of which is CTransPath, formulated by Wang et al. It works by combining a convolutional neural network for image processing and a Swin Transformer as a self-supervised hybrid CNN–Swin Transformer image encoder with pre-learning, via semantically relevant contrast learning by pairing instances with matching relevance scores [69]. Phikon is another model developed by Filiot et al. that employs a self-supervised ViT-Base image encoder, whereas Phikon-v2 uses a larger ViT architecture. Another line of work, represented by Neidlinger et al., benchmarked pathology foundation models as frozen feature extractors for weakly supervised computational pathology. In this framework, H&E WSIs are first tessellated into image patches, pretrained models are used to extract patch-level features, and downstream models then aggregate these features for biomarker, morphological, and prognostic prediction [70]. Within this category, UNI and Virchow are self-supervised pathology image encoders that primarily provide transferable patch-level representations for downstream tasks [71,72]. In contrast, Prov-GigaPath is designed to incorporate whole-slide context rather than serving only as a patch-level encoder. Developed by Xu et al. in collaboration with Microsoft, Providence, and the University of Washington. It introduces LongNet, which reduces the number of attention computations per vision transformer by using dilated self-attention instead of the quadratic standard self-attention, thereby preventing exceeding of the tile limit for each visual transformer owing to the large size of WSIs [73]. Vision–language and multimodal systems serve a different purpose. CONCH is a model developed by Lu et al. that supports image-text alignment, cross-modal retrieval, classification, and captioning [74]. PathChat is another model from Lu et al. that serves as an AI copilot to pathologists, providing visual and natural-language interaction for pathology image interpretation and question answering [75]. Together, these models illustrate the expanding landscape of computational pathology, from image encoders and whole-slide representation learning to image-text alignment and multimodal pathology assistance. Their use in CAF-related applications therefore depends on the specific task and validation setting. Table 2 summarizes representative pathology foundation models that may serve as candidate backbones for H&E-based stromal profiling. Most have not been directly validated for CAF-specific tasks.

### 5.4. Matching Model Architecture to Histopathology Tasks

CNNs, MIL frameworks, and pathology foundation models address different levels of WSI analysis and should be selected according to the prediction task, annotation availability, and interpretability requirement. CNNs are commonly used as local image encoders or supervised classifiers and are effective for patch- or region-level morphological recognition when adequate annotations are available, but they do not inherently model whole-slide heterogeneity or long-range spatial context. MIL frameworks are particularly suited to weakly supervised WSI tasks because they aggregate information from multiple tiles using slide-level labels. They can highlight model-relevant regions, but attention or contribution scores should be interpreted as model attribution rather than direct biological localization. Foundation models provide transferable pretrained representations and may reduce task-specific annotation requirements, especially in limited-data settings. However, their performance depends on the downstream task, training corpus, aggregation strategy, and external validation, and they are not automatically superior to smaller supervised encoders or interpretable morphology-based approaches. Therefore, CAF-related H&E modeling should benchmark multiple approaches, including CNN encoders, MIL variants, graph- or transformer-based models, foundation-model features, and hand-crafted spatial features, rather than prespecifying a single optimal architecture.

### 5.5. Stromal Segmentation and Nuclear Quantification on H&E

AI models that are particularly useful in stromal and cellular morphology assessment, via nuclear segmentation and tumor-stroma quantification, will also be explored. Nuclear segmentation isolates and quantifies nuclei to support cellular morphological assessment, but it does not itself determine fibroblast lineage or CAF subtype identity. An example of their use in the context of CAFs is the study by Leivonen et al., where their team used nuclear segmentation technology to support the characterization of CAF-associated stromal populations in samples of Hodgkin lymphoma to facilitate further workup to identify their subtypes [77]. Common examples of nuclear segmentation models include Cellpose, developed by Stringer et al., which uses a generalist deep-learning framework to segment cells and nuclei across diverse microscopy images. It is useful in heterogeneous or crowded cellular regions where conventional task-specific models may show reduced robustness or require extensive retraining [78]. Another common model is Hover-Net, developed by Graham et al., which utilizes vertical and horizontal distance maps between the center of the mass and nuclear pixels to separate clustered nuclei. Like StarDist, it is useful in crowded cells to segment nuclei as the mapping method is not affected by overlapping cells [79]. Applications of AI models for stromal assessment include tumor-stroma quantification, which serves as a prognostication tool by calculating the tumor-stroma ratio (TSR), which is a key prognosticator in colorectal cancer specimens. AI models that calculate the TSR were able to calculate and classify tumor specimens based on the TSR. In a study by Geessink et al., specimens were identified as “stroma high” and “stroma low” by both visual and automated methods. The results showed that automated TSR rather than visual TSR served as an independent prognostication tool [80]. In another study by Smit et al., automated TSR showed a strong correlation with visual TSR, suggesting its potential role in assisting pathologists [81]. These examples demonstrate AI-based stromal quantification and morphological assessment.

### 5.6. Spatial Profiling of the TME from H&E

In addition to cell identification, AI models are also useful in generating spatial atlases of the tumor microenvironment (TME). One example is the generation of a spatial atlas of tumor-infiltrating lymphocytes by Saltz et al., which utilized CNNs to locate TIL areas and affinity propagation to visualize their infiltration patterns across 13 TCGA samples [82]. This was extended to twenty-three TCGA samples in a similar study from Abousamra et al. [83]. Moreover, Zhang et al. developed another method to locate TILs through TILScout, an AI model that determines TIL density on WSI [84]. In addition to the technology itself, studies have shown its usefulness in prognostication. For instance, the study by AbdulJabbar et al. showcased the use of spatial atlases to map the distribution of immune and stromal cells in lung adenocarcinoma samples, and discovered that immune-cold regions, those with low lymphocyte density, had earlier diversification of tumor clones and a higher risk of relapse to treatment than immune-hot regions [85]. Another example demonstrating their prognostic value is demonstrated in a study by Trahearn et al., where spatial atlases of infiltrating vascular endothelial cells and TILs were independent predictors of progression-free survival in colorectal cancer [86]. Besides TILs, spatial atlases can also predict molecular phenotypes based on their analysis of WSIs. This is demonstrated by Diao et al., who discovered that human-interpretable features generated from AI models correlated with markers of the TME, which in turn predict molecular signatures of clinically relevant proteins [87]. Lastly, spatial atlases from AI models are also applicable to CAFs, as seen in the study by Markey et al., where their analysis via PathExplore revealed high predicted TGF-β-CAF signature scores associated with CAF-rich stromal regions in the tumor stroma of breast cancer specimens, and a low signature in tumor epithelium and inflamed stroma [68].

## 6. Predicting Cancer-Associated Fibroblast Biology and Immunotherapy Biomarkers from H&E: Current Evidence

### 6.1. H&E-Based Inference of CAF-Associated Molecular States

**Figure 2:**
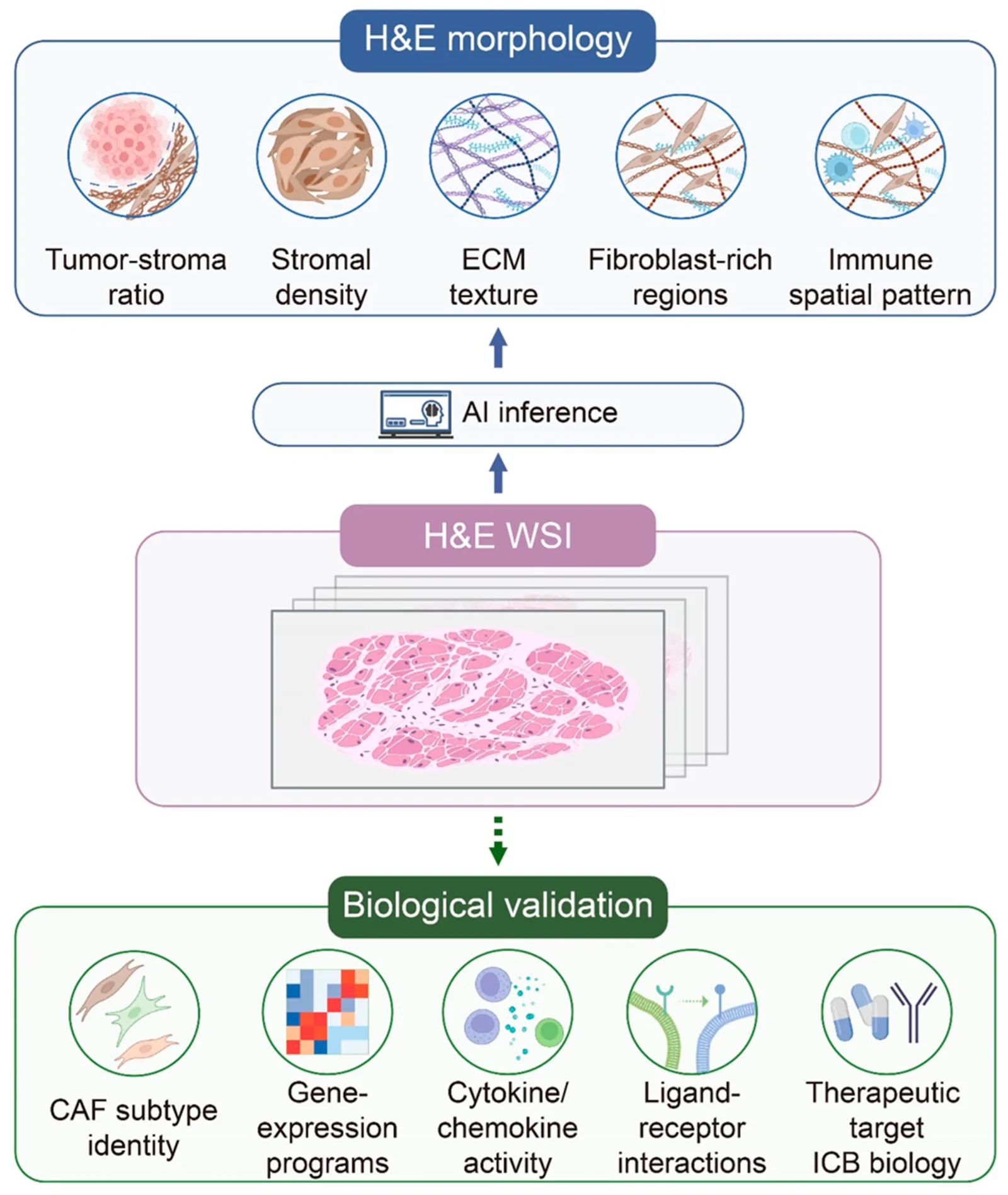
H&E-based CAF inference. Routine H&E whole-slide images provide morphological information on the tumor-stroma ratio, stromal density, ECM texture, fibroblast-rich regions, and immune spatial patterns. However, CAF subtype identity, gene-expression programs, cytokine and chemokine activity, ligand–receptor interactions, and therapeutic relevance require molecular or spatial validation. H&E-based artificial intelligence therefore serves as a scalable morphological screening layer rather than a standalone molecular classifier. AI-inferred H&E features should be interpreted with uncertainty, and molecular or spatial ground truth is typically available only for a validation subset of tissues.

Despite the heterogeneity of fibroblast subtypes, the recent literature has shown that it is feasible for H&E-based AI to infer CAF-associated spatial states using weak supervision from spatial transcriptomics, multiplex immunofluorescence (mIF), or imaging mass cytometry (IMC). Figure 2 provides a summary of what H&E WSIs can and cannot reveal about CAFs. As mentioned previously, Markey et al. demonstrated the use of aMIL to spatially predict TGF-β CAF gene signatures across various tumor types from H&E WSIs [68]. From a broader perspective, Fu et al. established that pan-cancer histological analysis can accurately predict mutations, tumor composition, and prognosis [88], which is corroborated by Schmauch et al. through their use of the deep-learning model HE2RNA in predicting RNA-seq expression from WSIs [89]. More recent examples include GHIST, developed by Fu et al., which links H&E images with spatial gene-expression prediction [90], and the Bell pancreatic cancer spatial integration, where they employed a semi-supervised learning framework [36]. However, CAF classifications across studies are derived from different tumor types, species, assay platforms, and clustering resolutions, and may represent partially overlapping or continuous cellular states rather than discrete classes. This context dependence creates substantial label uncertainty and remains a major challenge for cross-study harmonization and H&E-based prediction.

### 6.2. H&E-Derived Immunotherapy-Related Biomarkers

Recent advances have shown that immunotherapy biomarkers can be inferred from H&E using computational models. For instance, alongside the demonstration from Kather et al., Gustav et al. demonstrated the use of a deep-learning system to predict MSI and DNA polymerase ε (POLE) mutation status in colorectal tumors from H&E WSIs [91]. This trend continues, as evidenced by Saldanha et al., through the use of swarm learning AI models to predict BRAF mutation and MSI via H&E WSIs of colorectal tumors [92]. Moreover, Lazard et al. showed that AI can identify HRD-associated morphological patterns from breast H&E WSIs, suggesting a potential role for histology-based screening or enrichment rather than the replacement of established molecular assays [93]. Similarly, Shamai et al. had also illustrated the automated prediction of PD-L1 status from H&E WSIs [94]. Another important immunotherapy biomarker derived from H&E is tertiary lymphoid structure morphology [95], which were shown by Wang et al. to have a consistent association with improved ICB response [96], which is confirmed by van Rijthoven et al. using HookNet-TLS for TLS detection and prognostic assessment [97]. Collectively, these studies support the feasibility of deriving immunotherapy-relevant molecular and spatial features from H&E. However, these models should currently be regarded as screening, triage, or enrichment tools rather than replacements for established molecular assays, unless clinically acceptable performance, calibration, robustness, and prospective utility are demonstrated.

### 6.3. H&E-Based Prediction of ICB Response

Despite the increasing availability of AI in predicting biomarkers from H&E, there still appears to be a gap in the transition from computational pathology to real-life application in the clinical setting. Hu et al. demonstrated the use of deep learning in the prediction of the anti-PD-1 response of melanoma and NSCLC, but they reported that only a small subset of patients can benefit from ICB [98]. Similarly, the melanoma classifier developed by Johannet et al. [99], the multimodal radiology–pathology–genomics model for NSCLC reported by Vanguri et al. [100], and the subsequent multimodal extension reported by Captier et al. [101] primarily emphasized tumor-intrinsic, immune, radiological, pathological, or molecular features. Explicit stromal or CAF-associated spatial features were not separately characterized in these models, suggesting that the potential incremental value of CAF-aware spatial information remains to be evaluated in future ICB-treated cohorts. Representative histopathology-based and multimodal ICB-response models are summarized in Table 3.

Together, these studies illustrate the growing potential of histopathology-based and multimodal models for ICB-response prediction, while also highlighting substantial differences in input data, response definitions, and validation strategies. In this context, stromal and CAF-associated spatial features may provide complementary information beyond tumor-intrinsic and established biomarkers, including PD-L1, TMB, and MSI, but their incremental value should be tested in independent ICB-treated cohorts with prespecified comparisons against existing clinical and molecular predictors.

### 6.4. Hybrid Pipelines Combining Spatial Omics and H&E

Spatial omics is substantially more resource-intensive than routine H&E imaging, making a two-stage strategy more suitable for scalable CAF profiling. In a selected reference subset, paired spatial transcriptomics, mIF, or IMC can provide molecularly informed CAF-state annotations for model development and orthogonal validation. Once these reference relationships are established, the trained model can be applied to larger H&E-only cohorts to infer CAF-associated spatial states. Additional molecular data, such as bulk RNA sequencing, may further support biological interpretation, but are not required for every case. Such H&E-derived outputs should be regarded as inferred CAF-state maps rather than experimentally validated CAF subtype classifications. Such approaches should currently be regarded as a research framework, and whether they improve immunotherapy stratification requires validation in independent, clinically annotated ICB-treated cohorts.

## 7. A Proposed Translational Roadmap for H&E-Based CAF Spatial Inference and Immunotherapy Stratification

### 7.1. Multimodal Training and the Construction of an H&E-Fibroblast Rosetta Stone

The first step of model training is to establish a multi-institutional pan-cancer cohort design. The cohorts that are selected must be representative on a broad scale, thus requiring the integration of the internationally reputable Cancer Genome Atlas, our local gastric, colorectal and hepatocellular cohorts from the Chinese University of Hong Kong, and specific immune checkpoint inhibitor clinical-trial cohorts. Rather than requiring all modalities for every case, the cohort should be organized in a tiered manner. Discovery and development cohorts would primarily provide H&E WSIs and relevant clinicopathological information as the minimum viable dataset, with documented ICB outcomes included when treatment-response modeling is intended. A selected orthogonal reference subset would pair H&E with spatial transcriptomics (e.g., Visium HD or Xenium), mIF, or IMC to provide molecularly informed CAF-state reference maps rather than definitive pixel-level ground truth [102,105]. Because spatial-omics labels may be affected by spot mixing, differences in resolution, transcript dropout, platform-specific marker sensitivity, tissue deformation, registration error, and segmentation uncertainty, these reference maps should be treated as probabilistic and uncertainty-aware annotations for biological validation and weak supervision. Bulk RNA sequencing and other molecular data would serve as optional enrichment rather than mandatory inputs. Independent external clinical-evaluation cohorts would primarily require routine H&E WSIs and documented treatment outcomes. Foundation models (e.g., UNI or Virchow) combined with aMIL represent one candidate modeling strategy rather than necessary architecture. Given the variable performance of pathology foundation models across tasks and cohorts, candidate approaches should be comparatively evaluated, including alternative MIL frameworks, supervised encoders, transformer- or graph-based models, and interpretable morphology-based spatial features.

To facilitate cross-study model development, we propose a provisional five-state harmonization framework comprising myCAF, iCAF, apCAF, matCAF, and LRRC15-positive CAF programs, informed by the Cords 2023 [25], Chen 2023 [13], and Galbo 2021 [20] classifications. This framework is intended as an operational mapping strategy rather than a definitive or mutually exclusive biological taxonomy. Overlap between states, particularly between LRRC15-positive and myofibroblastic or matrix-associated programs, should therefore be expected. The proposed framework should be regarded as testable and will require cross-tumor, cross-platform, and functional validation before it can be adopted as a standardized label space for computational modeling.

### 7.2. Spatial Fibroblast Signature Extraction

The next step is to ensure that the trained model can infer CAF-associated spatial states in relation to the provisional harmonization framework proposed above. Given the potential overlap and continuity among CAF states, probabilistic or multi-state representations may be more appropriate than enforcing strictly mutually exclusive labels. Spatial transcriptomics, mIF, or IMC-derived annotations should therefore be treated as molecularly informed reference labels rather than definitive pixel-level ground truth, because spot mixing, tissue deformation, registration error, segmentation uncertainty, transcript dropout, platform-specific sensitivity, batch effects, and discordance between transcriptional and protein states may affect label reliability. Accordingly, H&E–molecular co-registration should include quality control, and regions with poor alignment, low molecular quality, or unreliable segmentation should be excluded or down-weighted. CAF-state assignment should use probabilistic labels, confidence scores, or region-level supervision, rather than assuming exact pixel-level subtype boundaries.

A panel of interpretable spatial features must be clearly defined and be able to be computed from the trained model in order to allow for proper inference by the model. Examples of these spatial features include predicted fibroblast-rich density per square millimeter, inferred CAF–T cell minimum and mean distances, fibroblast-tumor interface morphology, CAF-state spatial entropy, collagen alignment angle as recovered from H&E texture, and cellular-neighborhood composition derived from the Schürch et al. cellular-neighborhood framework [106]. These predicted features should be validated on held-out tissues using an orthogonal assay when feasible. The goal of this phase is to produce a feature vector per slide that can subsequently be evaluated for reproducibility across institutions and potential integration into clinical informatics systems. To ensure quality control of the model, the regulator and practicing pathologist must be able to inspect what the model attends to, and be able to make their own judgements over ambiguous cases.

### 7.3. Stratification Model Training and Patient-Level Validation

Afterwards, the goal is to translate the results from bench to bedside by training a survival or response classification model that can map the feature vector mentioned above to immunotherapy outcome. Before model training, the clinical setting of the stratification task should be defined, including the target population, intended use, ICB regimen, treatment line, outcome definition, and prediction time point. For instance, a pre-treatment model for objective response to anti-PD-1 monotherapy should be distinguished from a model designed to estimate long-term survival after chemoimmunotherapy, because CAF-associated spatial features may have different predictive meanings across treatment settings and disease stages.

External validation is required on multiple independent centers, with explicit assessment of domain shift attributable to scanner, stain protocol and demographic composition. To reduce data leakage, data splitting should be performed at the patient level rather than the tile level. Whenever possible, patients, slides, tissue blocks, and institutions should be separated across training, validation, and test partitions. Model selection and hyperparameter tuning should be conducted within nested validation procedures, and the final model should be evaluated only once in a locked test set or independent external cohort. For foundation-model-based pipelines, potential overlap between pretraining datasets and evaluation cohorts should also be assessed and minimized where feasible.

Cohort design should account for the number of outcome events, responder and non-responder balance, and model complexity. Missing clinical, imaging, pathological, or molecular variables should be handled using prespecified strategies rather than post hoc complete-case exclusion alone. Technical variation introduced by staining protocols, scanners, tissue processing, and institutional workflows should be addressed through stain normalization, scanner harmonization, batch-aware training, or sensitivity analyses. Importantly, retrospective external validation should be viewed as an intermediate step rather than sufficient evidence for clinical implementation. Prospective validation in clinically annotated cohorts, ideally including predefined endpoints and locked model parameters, is needed to determine whether CAF-associated H&E features remain reproducible and clinically informative in real-world workflows. These safeguards are necessary to ensure that patient-level predictions reflect reproducible CAF-associated spatial information rather than dataset-specific artifacts.

### 7.4. Clinical Evaluation and Biomarker Comparison

It is crucial to highlight that the new biomarker pertaining to fibroblast subtypes is not here to replace the already-established biomarkers, such as PD-L1 immunohistochemistry, tumor mutational burden, and microsatellite instability, but to add incremental value and information across prespecified subgroups. Accordingly, CAF-aware H&E models should be evaluated as complementary tools rather than standalone decision rules. Their performance should be compared with standard biomarkers and clinicopathological variables, including PD-L1, TMB, MSI, disease stage, treatment line, and performance status.

By comparing the new biomarker head-to-head and in-ensemble to established biomarkers, the aim is to evaluate whether CAF-associated spatial information can improve immunotherapy stratification. In addition to discrimination metrics such as AUC, C-index, sensitivity, and specificity, model evaluation should include calibration intercept, calibration slope, calibration curves, and uncertainty intervals. Decision-curve analysis can further determine whether model-guided stratification provides net clinical benefit over existing clinical and molecular predictors. Subgroup performance sho uld also be assessed across sex, ancestry, tumor type, institution, treatment regimen, and other clinically relevant strata. After locked external evaluation, prospective clinical-impact studies are needed to determine whether model-guided patient selection, treatment allocation, or combination-treatment design can improve clinical outcomes.

### 7.5. Companion-Diagnostic Translation

Subsequently, clinical deployment would require careful consideration of the model’s intended use, clinical claim, and implementation setting. A CAF-aware H&E model used for exploratory biomarker discovery should be distinguished from clinician-facing decision-support software, software as a medical device, and an in vitro diagnostic or companion-diagnostic tool for treatment selection. Locked algorithms should also be distinguished from adaptive models, because model updates, retraining, and post-deployment changes may require different validation and regulatory considerations. In addition, regulatory pathways differ across jurisdictions, including FDA pathways in the United States and CE marking or IVDR-related requirements in Europe. Regulatory terms, including clearance, authorization, approval, qualification, and Breakthrough Device Designation, should not be used interchangeably, and unsupported regulatory precedents should be avoided. In practice, implementation would also require digital scanning, quality control, slide storage, model-version monitoring, integration with pathology information systems, and a pathologist-in-the-loop workflow. Broader ethical, regulatory, legal, and patient data governance challenges are discussed in the following sections.

### 7.6. Pitfalls and Mitigation

Despite the promising outcomes, a few principal obstacles remain to be tackled. The major obstacle arises from fibroblast plasticity due to tissue remodeling under immunotherapy. To deal with this issue, longitudinal sampling on treatment is the ideal choice. Another challenge is the absence of a canonical CAF reference across tumor types and experimental platforms. Rather than being fully resolved by a fixed taxonomy, this limitation may be mitigated through flexible state mapping and cross-tumor, cross-platform, and functional validation of the proposed harmonization framework. The size and representativeness of the CAF validation subset will also be important, because a limited molecularly annotated cohort may not capture the full spectrum of CAF heterogeneity across tumor types and institutions. Methodological safeguards are also essential for reliable patient-level prediction, including prespecified outcome definitions, patient-level rather than tile-level splitting, separation of patients, slides, blocks, and institutions across partitions, nested model tuning, prevention of pretraining–evaluation overlap, predefined handling of missing data, stain/scanner harmonization, locked external evaluation, calibration assessment, decision-curve analysis, comparison with standard biomarkers, subgroup assessment, and prospective clinical-impact evaluation.

There is also a demographic bias from the underrepresentation of Asian, African, and Latin American patients in foundation-model training corpora, which can be partly mitigated through broader multi-institutional and geographically diverse validation, including Asian representation from our institution. In addition, pan-cancer CAF-aware modeling should not be assumed to be optimal by default, because a shared CAF label does not necessarily imply a shared relationship between CAF spatial organization and ICB response across cancer types. Pan-cancer models should therefore be compared with tumor-specific, cancer-type-stratified, and hierarchical or multitask alternatives. 

**Figure 3:**
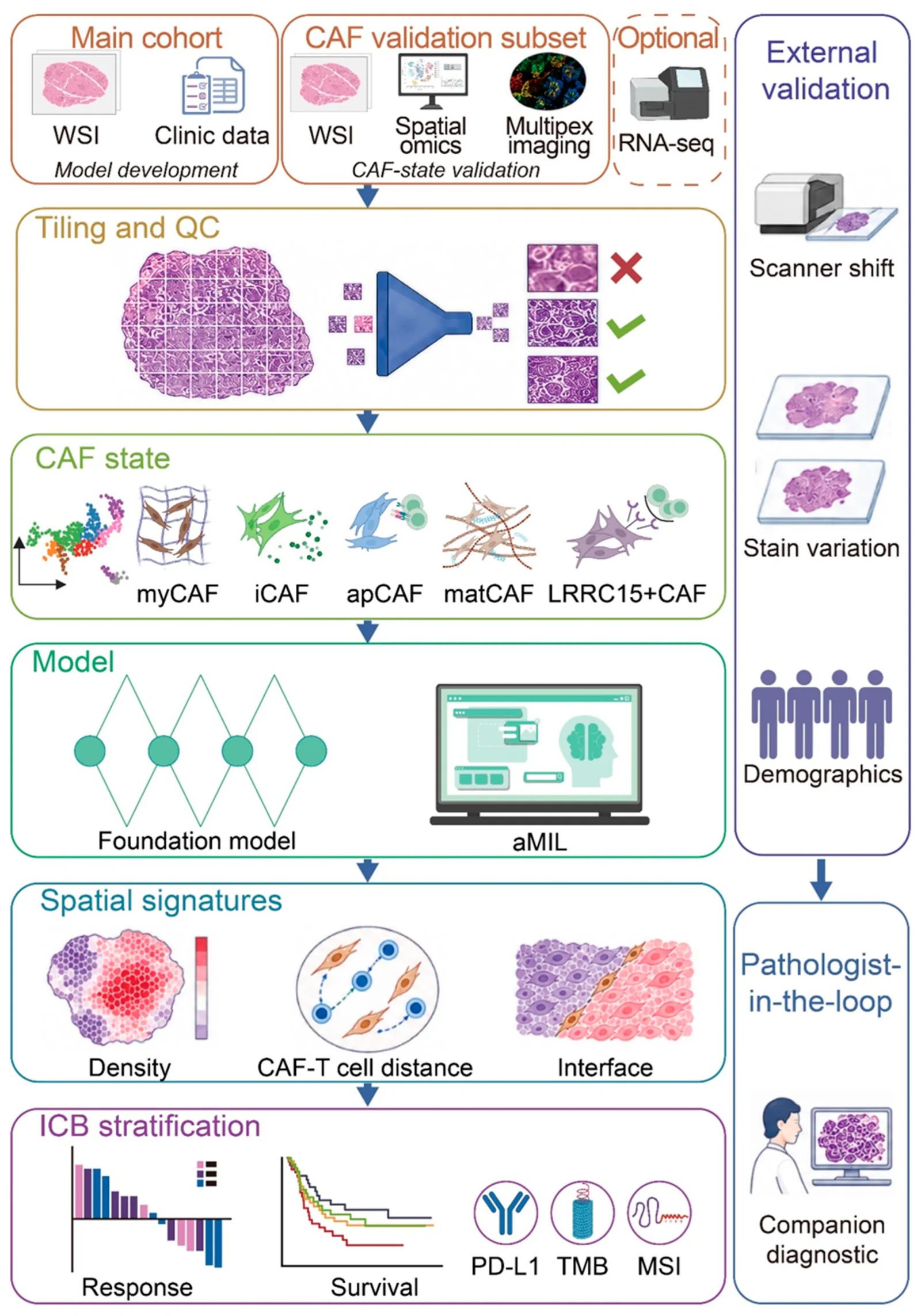
Proposed framework for H&E-based CAF spatial profiling and immunotherapy stratification. The main cohort provides H&E WSIs and clinical data for model development, while a selected CAF validation subset incorporates spatial omics or multiplex imaging for CAF-state annotation and validation; bulk RNA sequencing serves as optional molecular support. Following tiling and quality control, provisional CAF states are integrated into candidate computational models to infer spatial features, including CAF-rich density, CAF–T cell distance, and tumor-stroma interfaces. These features are subsequently evaluated for ICB response and survival stratification alongside established biomarkers, including PD-L1, TMB, and MSI. External validation assesses scanner, staining, and demographic variation before potential pathologist-in-the-loop clinical translation.

Another boundary of the proposed roadmap is its dependence on available histological tissue. The H&E-based CAF spatial profiling framework is most directly applicable to tumor types and clinical settings in which diagnostic biopsy, surgical resection, or archival pathology material is available. In tumors that are frequently diagnosed or monitored primarily by radiological imaging, such as some HCC cases, routine H&E material may be unavailable for patient-level inference. In these settings, radiology- or radiomics-based models may be required as complementary strategies, although they capture macroscopic imaging phenotypes rather than microscopic CAF states. Future studies with paired radiology, pathology, and spatial-omics data could explore cross-modal integration. Figure 3 provides a summary of our computational pipeline.

## 8. Future Directions and Open Challenges

### 8.1. Next Wave of Multimodal Foundation Models

The future of computational pathology is evidenced by the transition from passive prediction to agent-based pathology copilots. The new age of multimodal foundation models will aim to align H&E WSIs with spatial omics and clinical information from electronic health records, as exemplified by TITAN, MUSK, OmiCLIP and mSTAR [76,107,108,109].

### 8.2. Longitudinal Stromal Dynamics Under Immunotherapy

With tissue remodeling under immunotherapy as one of the major obstacles, dynamic biomarkers are the future direction, where stromal remodeling can be captured instead of the baseline state. Pre-treatment and on-treatment biopsies, complemented by liquid biopsy, can be pursued to allow for the training of dynamic biomarkers.

### 8.3. Maturation of Biology-Informed AI

With the improvement of the quality of biology-informed AI, where interpretability is key for quality control, the trajectory of AI must slowly but surely move from black-box prediction towards mechanism-aware models that explicitly learn fibroblast-T cell distance, collagen alignment, and cytokine gradient as latent features.

### 8.4. Equity and Global Deployment

The improvement in AI must also go hand-in-hand with the improvement in the equitable distribution of its usage, as stated in 2024’s *Nature Reviews Clinical Oncology* [110]. The broad availability of H&E slides may facilitate wider implementation once appropriate digitization, storage, quality-control, and validation infrastructure is available, while complementary molecular profiling can support model development and validation where feasible. An important pre-requisite is that training cohorts should be demographically representative in order to alleviate health disparities.

### 8.5. Patient Data Governance and Representation

The development of H&E-based AI models requires digitized pathology slides linked to clinical, molecular, treatment, and demographic data. Such data should be used under appropriate institutional approval, consent or consent-waiver procedures, de-identification, secure storage, and controlled access. A key ethical concern is population representation. If training cohorts underrepresent certain ancestry groups, geographic regions, institution types, or treatment settings, model performance may be less reliable in these populations and may worsen existing disparities.

### 8.6. Regulatory and Legal Translation

Clinical deployment of CAF-aware H&E models also requires regulatory and legal consideration. Models used for exploratory biomarker discovery should be distinguished from clinician-facing decision-support tools, software as a medical device, or companion-diagnostic tests for treatment selection. Regulatory evaluation should consider intended use, validation design, data provenance, model versioning, cybersecurity, audit trails, post-deployment monitoring, and human oversight. Legal responsibility should also be clarified when AI outputs influence diagnosis or treatment decisions.

## 9. Limitations of This Review

Several considerations should be kept in mind when interpreting this review. First, this article was designed as a narrative review with a structured literature search rather than a systematic review, and therefore did not include formal evidence grading or quantitative synthesis. Although we aimed to include representative and relevant studies across CAF biology, immunotherapy, spatial omics, and computational pathology, potential selection bias, citation bias, and publication bias toward positive AI findings cannot be fully excluded. Second, the evidence discussed here is heterogeneous across cancer types, experimental platforms, and study designs, and includes both human and preclinical studies. These differences limit direct comparison across studies and may influence the generalizability of CAF-related conclusions. Third, direct studies linking CAF-specific H&E-derived features to ICB response remain scarce, and many computational pathology models still have limited external validation. Comparisons among pathology foundation models may also evolve rapidly as new models, datasets, and training strategies emerge. Finally, possible overlap between public datasets, foundation-model training corpora, and downstream evaluation cohorts remains difficult to fully exclude. Accordingly, the roadmap proposed in this review should be viewed as a research agenda for developing and validating CAF-aware H&E biomarkers, rather than as evidence of established clinical utility.

## 10. Conclusions

This review has formulated a conceptual translational roadmap that extends from H&E WSIs to a CAF-associated spatial atlas, with potential relevance to future immunotherapy stratification. Our proposed roadmap integrates multi-institutional cohorts, spatially resolved molecular references, and computational pathology models to extract interpretable CAF-associated spatial signatures from routine histology. Rather than relying on a fixed model architecture or definitive pixel-level CAF labels, this framework emphasizes tiered multimodal validation, uncertainty-aware spatial annotation, and patient-level evaluation. We further propose that these spatial signatures may be linked to immunotherapy outcomes and assessed alongside established biomarkers, including PD-L1, tumor mutational burden, and microsatellite instability. At present, this roadmap should be viewed as a research framework for developing and validating CAF-aware H&E biomarkers, rather than as evidence of an immediately deployable clinical tool. Current evidence primarily supports its biological rationale and computational feasibility, while its clinical utility remains to be established through rigorous external validation and prospective evaluation. Importantly, CAF-aware computational pathology is not intended to replace existing biomarkers, but may provide a complementary stromal–spatial layer for future immunotherapy stratification.

## Figures and Tables

**Table 1:** Representative CAF states and immune associations across study contexts.

CAF State	Tumor and Assay Context	Markers	Function and Location	Evidence Context	Reported Association
myCAF [39]	Breast cancer; mouse models with supporting human CAF analyses; immunostaining and transcriptomic assessment	α-SMA^high^IL-6^low^, *RGS5*; *MRC2* or Endo180 in the breast cancer myCAF context	Contractile and matrix-remodeling program; often enriched at tumor borders or fibroblast-rich stromal regions	Preclinical mechanistic evidence related to immune checkpoint blockade	Endo180-positive myCAF-related remodeling was associated with CD8+ T cell exclusion. Endo180 deletion reduced an α-SMA-positive CAF subset, increased CD8+ T cell infiltration, and improved response to immune checkpoint blockade in mouse models.
iCAF [17,40]	Breast cancer and NSCLC-related analyses; human single-cell or CAF-state analysis, functional immune assays, and chemoimmunotherapy-associated models	α-SMA^low^IL-6^high^, PDGFR, *CXCL12*, Ly6c1^high^, *FBLN1*; *Ly6c1* as a mouse-associated marker	Cytokine- and chemokine-secreting inflammatory program; usually located in interstitial or immune-cell-associated stromal regions	Context-dependent immune association; treatment-related evidence varies across tumor settings	iCAF-related programs should not be assigned one fixed immune phenotype. Inflammatory CAF programs may shape immune infiltration and cytokine signaling, while IL-6-related inflammatory stromal programs have also been linked to myeloid reprogramming and chemoimmunotherapy resistance in NSCLC.
apCAF [27,41]	Pancreatic cancer and glioma; human and mouse single-cell or multi-omics analyses	MHC-II genes, *CD74*; *HLA-DRA* and *HLA-DRB1* in human datasets; AQP4 reported in a glioma-specific apCAF context	Antigen-presentation-related fibroblast program; located near tumor nests, immune niches, or tumor-stroma interfaces depending on tumor context	Mechanistic immune modulation and observational immune-resistance association	In pancreatic cancer, mesothelial cell-derived apCAFs were linked to CD4+ T cell and Treg-related immune modulation. In glioma, apCAF–M2 macrophage interactions and AQP4-positive apCAF features were associated with immune resistance and poor prognosis.
vCAF [42]	Triple-negative breast cancer; human single-cell analysis, spatial transcriptomics, mIF, and neoadjuvant immunotherapy samples	*MCAM*, IL-6; CA9 reported in a TNBC-specific CAF–myeloid axis	Vascular-associated or hypoxia-related stromal program; often localized around perivascular or stromal–myeloid regions	Human spatial multi-omics and treatment-related observational evidence	CA9+ CAFs were reported to co-localize and interact with SPP1-positive TAMs, forming a stroma–myeloid axis associated with poor prognosis, reduced effector T cell infiltration, and attenuated response to neoadjuvant immunotherapy in TNBC.
matCAF [43]	HCC; human single-cell datasets, spatial validation, and ICB-treated patient cohort	*POSTN, COL5A1*, FAP in the HCC matCAF-FAP context	Matrix-producing and collagen-remodeling program; enriched in interstitial or peripheral stromal regions	Human ICB-response association with spatial and immune-correlative evidence	POSTN-positive or matCAF-FAP-related CAF programs were enriched in non-responders to anti-PD-1 therapy and were associated with reduced CD8+ T cell infiltration, Treg and M2-like macrophage enrichment, and SPP1-positive macrophage interactions.
LRRC15+ CAF [31,32]	Pancreatic cancer and multiple solid tumors; human single-cell analysis, immunotherapy-treated cohorts, and mouse functional models	*LRRC15*; TGFβ-activated myofibroblast-like program	Myofibroblastic and matrix-associated stromal program; often enriched around tumor islets or desmoplastic stromal regions	Human ICB-response association with preclinical functional validation	Elevated LRRC15+ CAF signatures were associated with poor response to anti-PD-L1 therapy across immunotherapy clinical-trial cohorts. Functional studies further showed that LRRC15+ myofibroblasts can suppress CD8+ T cell activity and influence anti-PD-L1 response in mouse models.
ecm-myCAF/CAF-S1 cluster 0 [17]	Breast cancer, melanoma, and NSCLC-related analyses; human single-cell analysis, flow cytometry, functional assays, and ICB-treated cohorts	*SDC1* and ECM-related genes	ECM-remodeling CAF-S1 program; enriched in tumor-associated stromal regions	Human immune-functional and ICB-response association	ecm-myCAF was reported to induce immunosuppressive Treg-associated programs, including increased PD-1 and CTLA4 protein expression in Tregs, and was associated with non-response to immunotherapy in CAF-S1 analyses.
TGF-β-myCAF/CAF-S1 cluster 3 [17]	Breast cancer, melanoma, and NSCLC-related analyses; human single-cell analysis, flow cytometry, functional assays, and ICB-treated cohorts	*LAMP5* and TGFβ-related program	TGFβ-associated myofibroblastic program; located in tumor-associated or adjacent stromal regions depending on tissue context	Human immune-functional and ICB-response association	TGFβ-myCAF was linked to Treg-associated immunosuppressive programs and was enriched in non-responder contexts together with ecm-myCAF and wound-myCAF. This supports a treatment-context-specific association rather than a universal CAF immune phenotype.

**Table 2:** Candidate foundation models for stromal profiling.

Model	Architecture	Pretraining	Access	Evaluation	Stromal Relevance
CTransPath [69]	CNN–Swin patch encoder; 28 M parameters	SRCL on TCGA and PAIP histology patches	Public code and weights; reuse according to repository terms	Patch retrieval, classification, weakly supervised WSI classification, mitosis detection, and gland segmentation; CAF-specific metric and CI not reported	Lightweight candidate backbone for stromal feature extraction; not a demonstrated CAF-profiling model
UNI [71]	ViT-L patch encoder; 307 M parameters	DINOv2 on more than 100 million H&E patches from more than 100,000 WSIs across 20 tissue types	Research-use code and weights available under model terms	Broad pathology benchmarks across classification, segmentation, retrieval, and slide-level tasks; CAF-specific metric and CI not reported	General-purpose backbone; CAF use requires downstream stromal labels, spatial omics, mIF, or MIL aggregation
Virchow [72]	ViT-H patch encoder; 632 M parameters	DINOv2 self-supervised learning on approximately 1.5 million H&E WSIs from approximately 100,000 patients at MSKCC	Model access available under provider terms	Pan-cancer, rare-cancer, and biomarker-related benchmarks; CAF-specific metric and CI not reported	High-capacity candidate backbone, but computational cost is higher and CAF relevance remains indirect
Prov-GigaPath [73]	Tile encoder plus LongNet slide encoder; patch-level and slide-level operation	Tile-level SSL and slide-level pretraining on 1.3 billion tiles from 171,189 slides, more than 30,000 patients, 28 cancer centers, and 31 tissue types	Open-weight model with public code and pretrained weights	Multiple slide-level prediction tasks; CAF-specific metric and CI not reported	Suitable for WSI-level stromal architecture and long-range tumor-stroma context, but not a validated CAF classifier
CONCH [74]	CoCa-based vision–language model with image and text encoders	Contrastive image–text learning and captioning on more than 1.17 million pathology image–caption pairs	Research-use model and code available under release terms	Image classification, retrieval, captioning, and segmentation benchmarks; CAF-specific metric and CI not reported	Useful for text-guided retrieval, weak annotation, and interpretability support; not a direct CAF quantification model
TITAN [76]	Multimodal whole-slide model with slide-level visual and language alignment	Visual SSL and vision–language alignment on 335,645 WSIs, pathology reports, and 423,122 synthetic captions	Academic research access available under model terms	Slide-level classification, retrieval, prognosis, and biomarker-related tasks; CAF-specific metric and CI not reported	Potential slide-level backbone for stromal-context modeling, but requires CAF-specific reference labels and external validation

**Table 3:** Representative models for ICB-response prediction from histopathology or multimodal data.

Study	Tumor and Treatment	Cohort and Response	Model Design	Validation and Performance	Stromal/CAF Assessment
Hu et al. [98]	Melanoma and lung cancer; anti-PD-1 therapy	Training set: 190 melanoma H&E slides; test sets: 54 melanoma samples and 55 lung cancer samples; Response was assessed by RECIST v1.1 in clinical cohorts; melanoma test set included responders/non-responders of approximately 15/21.	H&E WSI-based CNN.	Melanoma test AUC = 0.778, 95% CI 0.638–0.905; lung cancer test AUC = 0.645, 95% CI 0.494–0.784	No explicit stromal or CAF module
Johannet et al. [99]	Advanced melanoma; ICI therapy	Training cohort: 302 H&E slides; independent validation cohort: 40 slides; response assessed for ICI outcome; Class balance and assessment time were NR.	H&E-derived deep-learning features integrated with clinical variables; included tissue-compartment segmentation.	Independent validation; AUC = 0.800 on Aperio AT2 scans and AUC = 0.805 on Leica SCN400 scans	Tissue compartments segmented, but CAF-specific features not isolated
Vanguri et al. [100]	Advanced NSCLC; PD-(L)1 blockade	Training cohort: *n* = 247; Radiology validation cohort: *n* = 50; pathology validation cohort: *n* = 52; responders/non-responders = 62/185; response by RECIST v1.1 as CR/PR vs. SD/PD.	DyAM multimodal model integrating CT radiomics, digitized PD-L1 IHC pathology features, genomic data, and clinical information.	Tenfold cross-validation; multimodal AUC = 0.80, 95% CI 0.74–0.86; outperformed PD-L1 TPS and TMB.	Pathology input included, but stromal/CAF contribution was not separately evaluated.
Captier et al. [101]	Metastatic NSCLC; first-line pembrolizumab ± chemotherapy	Multimodal cohort: *n* = 317; subset with all modalities: *n* = 80; endpoints included OS, PFS, 1-year death, and 6-month progression.	Multimodal model integrating clinical data, PET/CT radiomics, digitized pathology slides, and bulk RNA-seq; multiple fusion strategies were evaluated.	Cross-validation; best 1-year death model AUC = 0.81 ± 0.03; best OS model C-index = 0.75 ± 0.01.	Pathomic features included inflammatory-cell proportion and spatial organization, but CAF-specific contribution was not isolated.
Li et al. [102]	NSCLC; ICI-treated cohort for response prediction	HEX was trained and validated on 819,000 histopathology tiles with matched 40-marker protein expression from 382 tumor samples. ICI-response prediction was evaluated in a separate cohort of 148 patients.	HEX generated virtual spatial proteomics from H&E, followed by MICA integration of original H&E and AI-derived virtual spatial proteomic profiles.	Independent technical and clinical validation; HEX-enabled integration improved immunotherapy-response prediction by 24–39% over conventional clinicopathological and molecular biomarkers; calibration NR.	Spatial immune and stromal cell-state features were evaluated, including stromal marker combinations, but CAF-specific incremental value was not isolated.
Rakaee et al. [103]	Advanced NSCLC; anti-PD-(L)1 monotherapy; first or subsequent treatment lines	*n* = 685; discovery cohort *n* = 446, validation cohort *n* = 239. Endpoints included ORR, PFS, and OS; ORR in validation cohort was available for 101/239 patients.	Supervised ML-based H&E analysis to quantify tumor cells, stromal cells, and TILs; PD-L1 and TMB were assessed separately.	Multicenter discovery-validation design; in the PD-L1-negative subgroup, TIL AUC = 0.77 versus TMB AUC = 0.65 for ICI-response classification; calibration NR.	Explicit stromal and TIL quantification was included, but no CAF-specific module was used.
Rakaee et al. [104]	Advanced or metastatic NSCLC; ICI monotherapy	*n* = 958; US development cohort *n* = 614, EU external validation cohort *n* = 344. ORR was defined by RECIST v1.1; ORR was 26% in development and 28% in validation cohorts.	Deep-IO, a supervised H&E WSI-based deep-learning model for ICI-response prediction; compared with PD-L1, TMB, and TILs.	Internal test AUC = 0.75, 95% CI 0.64–0.85; external validation AUC = 0.66, 95% CI 0.60–0.72; Deep-IO + PD-L1 AUC = 0.70, 95% CI 0.63–0.76; calibration NR.	Model was not CAF-specific; stromal contribution was not isolated as an independent predictor.

## Data Availability

No new data were created or analyzed in this study.

## Abbreviations

ABMIL	Attention-based multiple-instance learning
AI	Artificial intelligence
aMIL	Additive multiple-instance learning
apCAF	Antigen-presenting cancer-associated fibroblast
AQP4	Aquaporin 4
AUC	Area under the curve
CAF	Cancer-associated fibroblast
CAF-S1	Cancer-associated fibroblast subset 1
CAR-T	Chimeric antigen receptor T cell
CD4	Cluster of differentiation 4
CD8	Cluster of differentiation 8
CD74	Cluster of differentiation 74
CNN	Convolutional neural network
COL5A1	Collagen type V alpha 1 chain
COL11A1	Collagen type XI alpha 1 chain
CTLA-4	Cytotoxic T-lymphocyte-associated protein 4
CXCL9	C-X-C motif chemokine ligand 9
CXCL10	C-X-C motif chemokine ligand 10
CXCL12	C-X-C motif chemokine ligand 12
CXCR4	C-X-C motif chemokine receptor 4
DINOv2	Self-supervised vision transformer model
DPP4	Dipeptidyl peptidase 4
ECM	Extracellular matrix
FDA	Food and Drug Administration
FAP	Fibroblast activation protein
FBLN1	Fibulin 1
GPU	Graphics processing unit
H&E	Hematoxylin and eosin
HCC	Hepatocellular carcinoma
HE2RNA	H&E-to-RNA prediction model
HEST	Histology and spatial transcriptomics
HRD	Homologous recombination deficiency
ICB	Immune checkpoint blockade
IF	Immunofluorescence
IL-1	Interleukin-1
IL-2	Interleukin-2
IL-6	Interleukin-6
IMC	Imaging mass cytometry
iCAF	Inflammatory cancer-associated fibroblast
JAK/STAT	Janus kinase/signal transducer and activator of transcription
LAMP5	Lysosomal-associated membrane protein family member 5
LLM	Large language model
LRRC15	Leucine-rich repeat containing 15
matCAF	Matrix-producing cancer-associated fibroblast
MCAM	Melanoma cell adhesion molecule
MDSC	Myeloid-derived suppressor cell
MHC-II	Major histocompatibility complex class II
MIL	Multiple instance learning
mIF	Multiplex immunofluorescence
MSI	Microsatellite instability
myCAF	Myofibroblastic cancer-associated fibroblast
NADPH	Nicotinamide adenine dinucleotide phosphate
NF-κB	Nuclear factor kappa B
NK	Natural killer
NKG2A	Natural killer group 2A
NOX1	NADPH oxidase 1
NOX4	NADPH oxidase 4
NSCLC	Non-small-cell lung cancer
PD-1	Programmed cell death protein 1
PD-L1	Programmed death-ligand 1
PDGFR	Platelet-derived growth factor receptor
POLE	DNA polymerase epsilon
RGS5	Regulator of G protein signaling 5
SaMD	Software as a medical device
SDC1	Syndecan 1
SPP1	Secreted phosphoprotein 1
SSL	Self-supervised learning
TAM	Tumor-associated macrophage
TCGA	The Cancer Genome Atlas
TGF-β	Transforming growth factor beta
THBS1	Thrombospondin 1
TIL	Tumor-infiltrating lymphocyte
TLS	Tertiary lymphoid structure
TMB	Tumor mutational burden
TME	Tumor microenvironment
Treg	Regulatory T cell
TSR	Tumor-stroma ratio
vCAF	Vascular cancer-associated fibroblast
ViT-B	Vision Transformer Base
ViT-L	Vision Transformer Large
WSI	Whole-slide image
YAP1	Yes-associated protein 1
α-SMA	Alpha-smooth muscle actin

## References

[1] Wolchok J.D., Chiarion-Sileni V., Gonzalez R., Rutkowski P., Grob J.J., Cowey C.L., Lao C.D., Wagstaff J., Schadendorf D., Ferrucci P.F. (2017). Overall Survival with Combined Nivolumab and Ipilimumab in Advanced Melanoma. N. Engl. J. Med..

[2] Reck M., Rodríguez-Abreu D., Robinson A.G., Hui R., Csőszi T., Fülöp A., Gottfried M., Peled N., Tafreshi A., Cuffe S. (2016). Pembrolizumab versus Chemotherapy for PD-L1-Positive Non-Small-Cell Lung Cancer. N. Engl. J. Med..

[3] Vuky J., Balar A.V., Castellano D., O’Donnell P.H., Grivas P., Bellmunt J., Powles T., Bajorin D., Hahn N.M., Savage M.J. (2020). Long-Term Outcomes in KEYNOTE-052: Phase II Study Investigating First-Line Pembrolizumab in Cisplatin-Ineligible Patients With Locally Advanced or Metastatic Urothelial Cancer. J. Clin. Oncol..

[4] Lenz H.J., Van Cutsem E., Luisa Limon M., Wong K.Y.M., Hendlisz A., Aglietta M., García-Alfonso P., Neyns B., Luppi G., Cardin D.B. (2022). First-Line Nivolumab Plus Low-Dose Ipilimumab for Microsatellite Instability-High/Mismatch Repair-Deficient Metastatic Colorectal Cancer: The Phase II CheckMate 142 Study. J. Clin. Oncol..

[5] Finn R.S., Qin S., Ikeda M., Galle P.R., Ducreux M., Kim T.Y., Kudo M., Breder V., Merle P., Kaseb A.O. (2020). Atezolizumab plus Bevacizumab in Unresectable Hepatocellular Carcinoma. N. Engl. J. Med..

[6] Kang Y.K., Boku N., Satoh T., Ryu M.H., Chao Y., Kato K., Chung H.C., Chen J.S., Muro K., Kang W.K. (2017). Nivolumab in patients with advanced gastric or gastro-oesophageal junction cancer refractory to, or intolerant of, at least two previous chemotherapy regimens (ONO-4538-12, ATTRACTION-2): A randomised, double-blind, placebo-controlled, phase 3 trial. Lancet.

[7] Mariathasan S., Turley S.J., Nickles D., Castiglioni A., Yuen K., Wang Y., Kadel E.E., Koeppen H., Astarita J.L., Cubas R. (2018). TGFβ attenuates tumor response to PD-L1 blockade by contributing to exclusion of T cells. Nature.

[8] Tauriello D.V.F., Palomo-Ponce S., Stork D., Berenguer-Llergo A., Badia-Ramentol J., Iglesias M., Sevillano M., Ibiza S., Cañellas A., Hernando-Momblona X. (2018). TGFβ drives immune evasion in genetically reconstituted colon cancer metastasis. Nature.

[9] Chakravarthy A., Furness A., Joshi K., Ghorani E., Ford K., Ward M.J., King E.V., Lechner M., Marafioti T., Quezada S.A. (2018). Pan-cancer deconvolution of tumor composition using DNA methylation. Nat. Commun..

[10] Sahai E., Astsaturov I., Cukierman E., DeNardo D.G., Egeblad M., Evans R.M., Fearon D., Greten F.R., Hingorani S.R., Hunter T. (2020). A framework for advancing our understanding of cancer-associated fibroblasts. Nat. Rev. Cancer.

[11] Bera K., Schalper K.A., Rimm D.L., Velcheti V., Madabhushi A. (2019). Artificial intelligence in digital pathology—New tools for diagnosis and precision oncology. Nat. Rev. Clin. Oncol..

[12] Shmatko A., Ghaffari Laleh N., Gerstung M., Kather J.N. (2022). Artificial intelligence in histopathology: Enhancing cancer research and clinical oncology. Nat. Cancer.

[13] Chen B., Chan W.N., Xie F., Mui C.W., Liu X., Cheung A.H.K., Lung R.W.M., Chow C., Zhang Z., Fang C. (2023). The molecular classification of cancer-associated fibroblasts on a pan-cancer single-cell transcriptional atlas. Clin. Transl. Med..

[14] Öhlund D., Handly-Santana A., Biffi G., Elyada E., Almeida A.S., Ponz-Sarvise M., Corbo V., Oni T.E., Hearn S.A., Lee E.J. (2017). Distinct populations of inflammatory fibroblasts and myofibroblasts in pancreatic cancer. J. Exp. Med..

[15] Elyada E., Bolisetty M., Laise P., Flynn W.F., Courtois E.T., Burkhart R.A., Teinor J.A., Belleau P., Biffi G., Lucito M.S. (2019). Cross-Species Single-Cell Analysis of Pancreatic Ductal Adenocarcinoma Reveals Antigen-Presenting Cancer-Associated Fibroblasts. Cancer Discov..

[16] Bartoschek M., Oskolkov N., Bocci M., Lövrot J., Larsson C., Sommarin M., Madsen C.D., Lindgren D., Pekar G., Karlsson G. (2018). Spatially and functionally distinct subclasses of breast cancer-associated fibroblasts revealed by single cell RNA sequencing. Nat. Commun..

[17] Kieffer Y., Hocine H.R., Gentric G., Pelon F., Bernard C., Bourachot B., Lameiras S., Albergante L., Bonneau C., Guyard A. (2020). Single-Cell Analysis Reveals Fibroblast Clusters Linked to Immunotherapy Resistance in Cancer. Cancer Discov..

[18] Costa A., Kieffer Y., Scholer-Dahirel A., Pelon F., Bourachot B., Cardon M., Sirven P., Magagna I., Fuhrmann L., Bernard C. (2018). Fibroblast Heterogeneity and Immunosuppressive Environment in Human Breast Cancer. Cancer Cell.

[19] Han C., Liu T., Yin R. (2020). Biomarkers for cancer-associated fibroblasts. Biomark. Res..

[20] Galbo P.M., Zang X., Zheng D. (2021). Molecular Features of Cancer-associated Fibroblast Subtypes and their Implication on Cancer Pathogenesis, Prognosis, and Immunotherapy Resistance. Clin. Cancer Res..

[21] Luo H., Xia X., Huang L.B., An H., Cao M., Kim G.D., Chen H.N., Zhang W.H., Shu Y., Kong X. (2022). Pan-cancer single-cell analysis reveals the heterogeneity and plasticity of cancer-associated fibroblasts in the tumor microenvironment. Nat. Commun..

[22] Foster D.S., Januszyk M., Delitto D., Yost K.E., Griffin M., Guo J., Guardino N., Delitto A.E., Chinta M., Burcham A.R. (2022). Multiomic analysis reveals conservation of cancer-associated fibroblast phenotypes across species and tissue of origin. Cancer Cell.

[23] Jia D., Liu Z., Deng N., Tan T.Z., Huang R.Y., Taylor-Harding B., Cheon D.J., Lawrenson K., Wiedemeyer W.R., Walts A.E. (2016). A COL11A1-correlated pan-cancer gene signature of activated fibroblasts for the prioritization of therapeutic targets. Cancer Lett..

[24] Cords L., Engler S., Haberecker M., Rüschoff J.H., Moch H., de Souza N., Bodenmiller B. (2024). Cancer-associated fibroblast phenotypes are associated with patient outcome in non-small cell lung cancer. Cancer Cell.

[25] Cords L., Tietscher S., Anzeneder T., Langwieder C., Rees M., de Souza N., Bodenmiller B. (2023). Cancer-associated fibroblast classification in single-cell and spatial proteomics data. Nat. Commun..

[26] Buechler M.B., Pradhan R.N., Krishnamurty A.T., Cox C., Calviello A.K., Wang A.W., Yang Y.A., Tam L., Caothien R., Roose-Girma M. (2021). Cross-tissue organization of the fibroblast lineage. Nature.

[27] Huang H., Wang Z., Zhang Y., Pradhan R.N., Ganguly D., Chandra R., Murimwa G., Wright S., Gu X., Maddipati R. (2022). Mesothelial cell-derived antigen-presenting cancer-associated fibroblasts induce expansion of regulatory T cells in pancreatic cancer. Cancer Cell.

[28] Burt R.J., Dey A., Akarca A., Allen H., Amerikanou R., Atkinson S., Auty D., Chatzigerou J., Cutler E., Guerra-Assuncao J.A. (2024). Mitochondrial dsRNA from B-ALL cells stimulates mesenchymal stromal cells to become cancer-associated fibroblasts. Blood Adv..

[29] Hosaka K., Yang Y., Seki T., Fischer C., Dubey O., Fredlund E., Hartman J., Religa P., Morikawa H., Ishii Y. (2016). Pericyte-fibroblast transition promotes tumor growth and metastasis. Proc. Natl. Acad. Sci. USA.

[30] Miyazaki Y., Oda T., Inagaki Y., Kushige H., Saito Y., Mori N., Takayama Y., Kumagai Y., Mitsuyama T., Kida Y.S. (2021). Adipose-derived mesenchymal stem cells differentiate into heterogeneous cancer-associated fibroblasts in a stroma-rich xenograft model. Sci. Rep..

[31] Dominguez C.X., Müller S., Keerthivasan S., Koeppen H., Hung J., Gierke S., Breart B., Foreman O., Bainbridge T.W., Castiglioni A. (2020). Single-Cell RNA Sequencing Reveals Stromal Evolution into LRRC15(+) Myofibroblasts as a Determinant of Patient Response to Cancer Immunotherapy. Cancer Discov..

[32] Krishnamurty A.T., Shyer J.A., Thai M., Gandham V., Buechler M.B., Yang Y.A., Pradhan R.N., Wang A.W., Sanchez P.L., Qu Y. (2022). LRRC15(+) myofibroblasts dictate the stromal setpoint to suppress tumor immunity. Nature.

[33] Biffi G., Oni T.E., Spielman B., Hao Y., Elyada E., Park Y., Preall J., Tuveson D.A. (2019). IL1-Induced JAK/STAT Signaling Is Antagonized by TGFβ to Shape CAF Heterogeneity in Pancreatic Ductal Adenocarcinoma. Cancer Discov..

[34] Croizer H., Mhaidly R., Kieffer Y., Gentric G., Djerroudi L., Leclere R., Pelon F., Robley C., Bohec M., Meng A. (2024). Deciphering the spatial landscape and plasticity of immunosuppressive fibroblasts in breast cancer. Nat. Commun..

[35] Hegde P.S., Chen D.S. (2020). Top 10 Challenges in Cancer Immunotherapy. Immunity.

[36] Bell A.T.F., Mitchell J.T., Kiemen A.L., Lyman M., Fujikura K., Lee J.W., Coyne E., Shin S.M., Nagaraj S., Deshpande A. (2024). PanIN and CAF transitions in pancreatic carcinogenesis revealed with spatial data integration. Cell Syst..

[37] Sathe A., Mason K., Grimes S.M., Zhou Z., Lau B.T., Bai X., Su A., Tan X., Lee H., Suarez C.J. (2023). Colorectal Cancer Metastases in the Liver Establish Immunosuppressive Spatial Networking between Tumor-Associated SPP1+ Macrophages and Fibroblasts. Clin. Cancer Res..

[38] Pantaleo J., Sjölund J., Bolivar P., Bocci M., Phung B., Malmberg M., Jönsson G.B., Pietras K. (2026). Spatial and temporal dynamics of cancer-associated fibroblast niches in breast cancer. Breast Cancer Res..

[39] Jenkins L., Jungwirth U., Avgustinova A., Iravani M., Mills A., Haider S., Harper J., Isacke C.M. (2022). Cancer-Associated Fibroblasts Suppress CD8+ T-cell Infiltration and Confer Resistance to Immune-Checkpoint Blockade. Cancer Res..

[40] Yang Y., Liu C., Yang L., Zheng S., Xu H., Zhang S., Tian L., Sun N., He J., Wang Y. (2026). IL-6 drives chemoimmunotherapy resistance in NSCLC by reprogramming myeloid cells and impairing cytotoxic lymphocyte function. Cancer Lett..

[41] Ren Y., Lu D., Wang F., Wang Z., Li J., Huang R., Lu Y., Duan A., Shou R., Liu J. (2025). A multi-omics atlas of CAF subtypes reveals apCAF-M2 macrophage interactions driving immune resistance in glioma. PLoS ONE.

[42] Ma Q., Wang J., Jiang Q. (2026). CA9+ cancer-associated fibroblasts cooperate with SPP1+ tumor-associated macrophages driving immune resistance in triple-negative breast cancer. Cell Mol. Life Sci..

[43] Wang H., Liang Y., Liu Z., Zhang R., Chao J., Wang M., Liu M., Qiao L., Xuan Z., Zhao H. (2024). POSTN(+) cancer-associated fibroblasts determine the efficacy of immunotherapy in hepatocellular carcinoma. J. Immunother. Cancer.

[44] Kuczek D.E., Larsen A.M.H., Thorseth M.L., Carretta M., Kalvisa A., Siersbæk M.S., Simões A.M.C., Roslind A., Engelholm L.H., Noessner E. (2019). Collagen density regulates the activity of tumor-infiltrating T cells. J. Immunother. Cancer.

[45] Pruitt H.C., Lewis D., Ciccaglione M., Connor S., Smith Q., Hickey J.W., Schneck J.P., Gerecht S. (2020). Collagen fiber structure guides 3D motility of cytotoxic T lymphocytes. Matrix Biol..

[46] Xiao Z., Todd L., Huang L., Noguera-Ortega E., Lu Z., Huang L., Kopp M., Li Y., Pattada N., Zhong W. (2023). Desmoplastic stroma restricts T cell extravasation and mediates immune exclusion and immunosuppression in solid tumors. Nat. Commun..

[47] Feig C., Jones J.O., Kraman M., Wells R.J., Deonarine A., Chan D.S., Connell C.M., Roberts E.W., Zhao Q., Caballero O.L. (2013). Targeting CXCL12 from FAP-expressing carcinoma-associated fibroblasts synergizes with anti-PD-L1 immunotherapy in pancreatic cancer. Proc. Natl. Acad. Sci. USA.

[48] Wong P.F., Wei W., Gupta S., Smithy J.W., Zelterman D., Kluger H.M., Rimm D.L. (2019). Multiplex quantitative analysis of cancer-associated fibroblasts and immunotherapy outcome in metastatic melanoma. J. Immunother. Cancer.

[49] Chen B., Tang H., Zheng X., Xie F., Yu P., Lyu Y., Feng T., Wu J., Liu J., Xu Y. (2025). Spatial and functional dissection of cancer-associated fibroblasts-mediated immune modulation in H. pylori-associated gastric cancer. Mol. Cancer.

[50] Chen X., Li Y., Huang J., Zhang Q., Tan C., Liu Y., Du Z. (2024). Prognosis and immunotherapy significances of a cancer-associated fibroblasts-related gene signature in bladder urothelial carcinoma. Discov. Oncol..

[51] Obradovic A., Graves D., Korrer M., Wang Y., Roy S., Naveed A., Xu Y., Luginbuhl A., Curry J., Gibson M. (2022). Immunostimulatory Cancer-Associated Fibroblast Subpopulations Can Predict Immunotherapy Response in Head and Neck Cancer. Clin. Cancer Res..

[52] Zhou L., Li Y., Zheng D., Zheng Y., Cui Y., Qin L., Tang Z., Peng D., Wu Q., Long Y. (2024). Bispecific CAR-T cells targeting FAP and GPC3 have the potential to treat hepatocellular carcinoma. Mol. Ther. Oncol..

[53] Waldhauer I., Gonzalez-Nicolini V., Freimoser-Grundschober A., Nayak T.K., Fahrni L., Hosse R.J., Gerrits D., Geven E.J.W., Sam J., Lang S. (2021). Simlukafusp alfa (FAP-IL2v) immunocytokine is a versatile combination partner for cancer immunotherapy. MAbs.

[54] Lan Y., Yeung T.L., Huang H., Wegener A.A., Saha S., Toister-Achituv M., Jenkins M.H., Chiu L.Y., Lazorchak A., Tarcic O. (2022). Colocalized targeting of TGF-β and PD-L1 by bintrafusp alfa elicits distinct antitumor responses. J. Immunother. Cancer.

[55] Storey C.M., Altai M., Lückerath K., Zedan W., Zhu H., Breuer L., Trajkovic-Arsic M., Park J., Hasson A., Siveke J. (2025). Development of a leucine-rich repeat-containing protein 15-targeted radio-immunotheranostic approach to deplete pro-tumorigenic mechanisms and immunotherapy resistance. Signal Transduct. Target Ther..

[56] Amengual J., Gonzalez-Sanchez E., Yáñez-Bartolome M., Sererols-Viñas L., Ravichandra A., Guiton C., Fuste N.P., Alay A., Hijazo-Pechero S., Martín-Mur B. (2025). NADPH oxidase 1/4 dual inhibition impairs transforming growth factor-beta protumorigenic effects in cholangiocarcinoma cancer-associated fibroblasts. Signal Transduct. Target Ther..

[57] Andtbacka R.H.I., Wang Y., Pierce R.H., Campbell J.S., Yushak M., Milhem M., Ross M., Niland K., Arbeit R.D., Parasuraman S. (2022). Mavorixafor, an Orally Bioavailable CXCR4 Antagonist, Increases Immune Cell Infiltration and Inflammatory Status of Tumor Microenvironment in Patients with Melanoma. Cancer Res. Commun..

[58] Campanella G., Hanna M.G., Geneslaw L., Miraflor A., Werneck Krauss Silva V., Busam K.J., Brogi E., Reuter V.E., Klimstra D.S., Fuchs T.J. (2019). Clinical-grade computational pathology using weakly supervised deep learning on whole slide images. Nat. Med..

[59] Lin S., Zhou H., Watson M., Govindan R., Cote R.J., Yang C. (2025). Impact of stain variation and color normalization for prognostic predictions in pathology. Sci. Rep..

[60] Ciga O., Xu T., Nofech-Mozes S., Noy S., Lu F.I., Martel A.L. (2021). Overcoming the limitations of patch-based learning to detect cancer in whole slide images. Sci. Rep..

[61] Coudray N., Ocampo P.S., Sakellaropoulos T., Narula N., Snuderl M., Fenyö D., Moreira A.L., Razavian N., Tsirigos A. (2018). Classification and mutation prediction from non-small cell lung cancer histopathology images using deep learning. Nat. Med..

[62] Kather J.N., Pearson A.T., Halama N., Jäger D., Krause J., Loosen S.H., Marx A., Boor P., Tacke F., Neumann U.P. (2019). Deep learning can predict microsatellite instability directly from histology in gastrointestinal cancer. Nat. Med..

[63] Ghaffari Laleh N., Muti H.S., Loeffler C.M.L., Echle A., Saldanha O.L., Mahmood F., Lu M.Y., Trautwein C., Langer R., Dislich B. (2022). Benchmarking weakly-supervised deep learning pipelines for whole slide classification in computational pathology. Med. Image Anal..

[64] Yao J., Zhu X., Jonnagaddala J., Hawkins N., Huang J. (2020). Whole slide images based cancer survival prediction using attention guided deep multiple instance learning networks. Med. Image Anal..

[65] Lu M.Y., Williamson D.F.K., Chen T.Y., Chen R.J., Barbieri M., Mahmood F. (2021). Data-efficient and weakly supervised computational pathology on whole-slide images. Nat. Biomed. Eng..

[66] Shi J., Sun D., Wu K., Jiang Z., Kong X., Wang W., Wu H., Zheng Y. (2025). Positional encoding-guided transformer-based multiple instance learning for histopathology whole slide images classification. Comput. Methods Programs Biomed..

[67] Li B., Li Y., Eliceiri K.W. (2021). Dual-stream Multiple Instance Learning Network for Whole Slide Image Classification with Self-supervised Contrastive Learning. Conf. Comput. Vis. Pattern Recognit. Work..

[68] Markey M., Kim J., Goldstein Z., Gerardin Y., Brosnan-Cashman J., Javed S.A., Juyal D., Pagidela H., Yu L., Rahsepar B. (2025). Spatial Mapping of Gene Signatures in Hematoxylin and Eosin-Stained Images: A Proof of Concept for Interpretable Predictions Using Additive Multiple Instance Learning. Mod. Pathol..

[69] Wang X., Yang S., Zhang J., Wang M., Zhang J., Yang W., Huang J., Han X. (2022). Transformer-based unsupervised contrastive learning for histopathological image classification. Med. Image Anal..

[70] Neidlinger P., El Nahhas O.S.M., Muti H.S., Lenz T., Hoffmeister M., Brenner H., van Treeck M., Langer R., Dislich B., Behrens H.M. (2026). Benchmarking foundation models as feature extractors for weakly supervised computational pathology. Nat. Biomed. Eng..

[71] Chen R.J., Ding T., Lu M.Y., Williamson D.F.K., Jaume G., Song A.H., Chen B., Zhang A., Shao D., Shaban M. (2024). Towards a general-purpose foundation model for computational pathology. Nat. Med..

[72] Vorontsov E., Bozkurt A., Casson A., Shaikovski G., Zelechowski M., Severson K., Zimmermann E., Hall J., Tenenholtz N., Fusi N. (2024). A foundation model for clinical-grade computational pathology and rare cancers detection. Nat. Med..

[73] Xu H., Usuyama N., Bagga J., Zhang S., Rao R., Naumann T., Wong C., Gero Z., González J., Gu Y. (2024). A whole-slide foundation model for digital pathology from real-world data. Nature.

[74] Lu M.Y., Chen B., Williamson D.F.K., Chen R.J., Liang I., Ding T., Jaume G., Odintsov I., Le L.P., Gerber G. (2024). A visual-language foundation model for computational pathology. Nat. Med..

[75] Lu M.Y., Chen B., Williamson D.F.K., Chen R.J., Zhao M., Chow A.K., Ikemura K., Kim A., Pouli D., Patel A. (2024). A multimodal generative AI copilot for human pathology. Nature.

[76] Ding T., Wagner S.J., Song A.H., Chen R.J., Lu M.Y., Zhang A., Vaidya A.J., Jaume G., Shaban M., Kim A. (2025). A multimodal whole-slide foundation model for pathology. Nat. Med..

[77] Leivonen S.K., Karihtala K., Pellinen T., Karjalainen-Lindsberg M.L., Aoki T., Steidl C., Leppä S. (2025). Characterization of cancer-associated fibroblasts and their spatial architecture reveals heterogeneity and survival associations in classic Hodgkin lymphoma. Hemasphere.

[78] Stringer C., Wang T., Michaelos M., Pachitariu M. (2021). Cellpose: A generalist algorithm for cellular segmentation. Nat. Methods.

[79] Graham S., Vu Q.D., Raza S.E.A., Azam A., Tsang Y.W., Kwak J.T., Rajpoot N. (2019). Hover-Net: Simultaneous segmentation and classification of nuclei in multi-tissue histology images. Med. Image Anal..

[80] Geessink O.G.F., Baidoshvili A., Klaase J.M., Ehteshami Bejnordi B., Litjens G.J.S., van Pelt G.W., Mesker W.E., Nagtegaal I.D., Ciompi F., van der Laak J. (2019). Computer aided quantification of intratumoral stroma yields an independent prognosticator in rectal cancer. Cell Oncol..

[81] Smit M.A., Ciompi F., Bokhorst J.M., van Pelt G.W., Geessink O.G.F., Putter H., Tollenaar R., van Krieken J., Mesker W.E., van der Laak J. (2023). Deep learning based tumor-stroma ratio scoring in colon cancer correlates with microscopic assessment. J. Pathol. Inf..

[82] Saltz J., Gupta R., Hou L., Kurc T., Singh P., Nguyen V., Samaras D., Shroyer K.R., Zhao T., Batiste R. (2018). Spatial Organization and Molecular Correlation of Tumor-Infiltrating Lymphocytes Using Deep Learning on Pathology Images. Cell Rep..

[83] Abousamra S., Gupta R., Hou L., Batiste R., Zhao T., Shankar A., Rao A., Chen C., Samaras D., Kurc T. (2021). Deep Learning-Based Mapping of Tumor Infiltrating Lymphocytes in Whole Slide Images of 23 Types of Cancer. Front. Oncol..

[84] Zhang H., Chen L., Li L., Liu Y., Das B., Zhai S., Tan J., Jiang Y., Turco S., Yao Y. (2025). Prediction and analysis of tumor infiltrating lymphocytes across 28 cancers by TILScout using deep learning. NPJ Precis Oncol..

[85] AbdulJabbar K., Raza S.E.A., Rosenthal R., Jamal-Hanjani M., Veeriah S., Akarca A., Lund T., Moore D.A., Salgado R., Al Bakir M. (2020). Geospatial immune variability illuminates differential evolution of lung adenocarcinoma. Nat. Med..

[86] Trahearn N., Sakr C., Banerjee A., Lee S.H., Baker A.M., Kocher H.M., Angerilli V., Morano F., Bergamo F., Maddalena G. (2025). Computational pathology applied to clinical colorectal cancer cohorts identifies immune and endothelial cell spatial patterns predictive of outcome. J. Pathol..

[87] Diao J.A., Wang J.K., Chui W.F., Mountain V., Gullapally S.C., Srinivasan R., Mitchell R.N., Glass B., Hoffman S., Rao S.K. (2021). Human-interpretable image features derived from densely mapped cancer pathology slides predict diverse molecular phenotypes. Nat. Commun..

[88] Fu Y., Jung A.W., Torne R.V., Gonzalez S., Vöhringer H., Shmatko A., Yates L.R., Jimenez-Linan M., Moore L., Gerstung M. (2020). Pan-cancer computational histopathology reveals mutations, tumor composition and prognosis. Nat. Cancer.

[89] Schmauch B., Romagnoni A., Pronier E., Saillard C., Maillé P., Calderaro J., Kamoun A., Sefta M., Toldo S., Zaslavskiy M. (2020). A deep learning model to predict RNA-Seq expression of tumors from whole slide images. Nat. Commun..

[90] Fu X., Cao Y., Bian B., Wang C., Graham D., Pathmanathan N., Patrick E., Kim J., Yang J.Y.H. (2025). Spatial gene expression at single-cell resolution from histology using deep learning with GHIST. Nat. Methods.

[91] Gustav M., Reitsam N.G., Carrero Z.I., Loeffler C.M.L., van Treeck M., Yuan T., West N.P., Quirke P., Brinker T.J., Brenner H. (2024). Deep learning for dual detection of microsatellite instability and POLE mutations in colorectal cancer histopathology. NPJ Precis Oncol..

[92] Saldanha O.L., Quirke P., West N.P., James J.A., Loughrey M.B., Grabsch H.I., Salto-Tellez M., Alwers E., Cifci D., Ghaffari Laleh N. (2022). Swarm learning for decentralized artificial intelligence in cancer histopathology. Nat. Med..

[93] Lazard T., Bataillon G., Naylor P., Popova T., Bidard F.C., Stoppa-Lyonnet D., Stern M.H., Decencière E., Walter T., Vincent-Salomon A. (2022). Deep learning identifies morphological patterns of homologous recombination deficiency in luminal breast cancers from whole slide images. Cell Rep. Med..

[94] Shamai G., Livne A., Polónia A., Sabo E., Cretu A., Bar-Sela G., Kimmel R. (2022). Deep learning-based image analysis predicts PD-L1 status from H&E-stained histopathology images in breast cancer. Nat. Commun..

[95] Deng S., Chen Y., Song B., Wang H., Huang S., Wu K., Chu Q. (2025). Tertiary lymphoid structures in cancer: Spatiotemporal heterogeneity, immune orchestration, and translational opportunities. J. Hematol. Oncol..

[96] Wang Q., Zhong W., Shen X., Hao Z., Wan M., Yang X., An R., Zhu H., Cai H., Li T. (2024). Tertiary lymphoid structures predict survival and response to neoadjuvant therapy in locally advanced rectal cancer. NPJ Precis Oncol..

[97] van Rijthoven M., Obahor S., Pagliarulo F., van den Broek M., Schraml P., Moch H., van der Laak J., Ciompi F., Silina K. (2024). Multi-resolution deep learning characterizes tertiary lymphoid structures and their prognostic relevance in solid tumors. Commun. Med..

[98] Hu J., Cui C., Yang W., Huang L., Yu R., Liu S., Kong Y. (2021). Using deep learning to predict anti-PD-1 response in melanoma and lung cancer patients from histopathology images. Transl. Oncol..

[99] Johannet P., Coudray N., Donnelly D.M., Jour G., Illa-Bochaca I., Xia Y., Johnson D.B., Wheless L., Patrinely J.R., Nomikou S. (2021). Using Machine Learning Algorithms to Predict Immunotherapy Response in Patients with Advanced Melanoma. Clin. Cancer Res..

[100] Vanguri R.S., Luo J., Aukerman A.T., Egger J.V., Fong C.J., Horvat N., Pagano A., Araujo-Filho J.A.B., Geneslaw L., Rizvi H. (2022). Multimodal integration of radiology, pathology and genomics for prediction of response to PD-(L)1 blockade in patients with non-small cell lung cancer. Nat. Cancer.

[101] Captier N., Lerousseau M., Orlhac F., Hovhannisyan-Baghdasarian N., Luporsi M., Woff E., Lagha S., Salamoun Feghali P., Lonjou C., Beaulaton C. (2025). Integration of clinical, pathological, radiological, and transcriptomic data improves prediction for first-line immunotherapy outcome in metastatic non-small cell lung cancer. Nat. Commun..

[102] Li Z., Li Y., Xiang J., Wang X., Yang S., Zhang X., Eweje F., Chen Y., Luo X., Li Y. (2026). AI-enabled virtual spatial proteomics from histopathology for interpretable biomarker discovery in lung cancer. Nat. Med..

[103] Rakaee M., Adib E., Ricciuti B., Sholl L.M., Shi W., Alessi J.V., Cortellini A., Fulgenzi C.A.M., Viola P., Pinato D.J. (2023). Association of Machine Learning-Based Assessment of Tumor-Infiltrating Lymphocytes on Standard Histologic Images With Outcomes of Immunotherapy in Patients With NSCLC. JAMA Oncol..

[104] Rakaee M., Tafavvoghi M., Ricciuti B., Alessi J.V., Cortellini A., Citarella F., Nibid L., Perrone G., Adib E., Fulgenzi C.A.M. (2025). Deep Learning Model for Predicting Immunotherapy Response in Advanced Non-Small Cell Lung Cancer. JAMA Oncol..

[105] Lin J.R., Chen Y.A., Campton D., Cooper J., Coy S., Yapp C., Tefft J.B., McCarty E., Ligon K.L., Rodig S.J. (2023). High-plex immunofluorescence imaging and traditional histology of the same tissue section for discovering image-based biomarkers. Nat. Cancer.

[106] Schürch C.M., Bhate S.S., Barlow G.L., Phillips D.J., Noti L., Zlobec I., Chu P., Black S., Demeter J., McIlwain D.R. (2020). Coordinated Cellular Neighborhoods Orchestrate Antitumoral Immunity at the Colorectal Cancer Invasive Front. Cell.

[107] Chen W., Zhang P., Tran T.N., Xiao Y., Li S., Shah V.V., Cheng H., Brannan K.W., Youker K., Lai L. (2025). A visual-omics foundation model to bridge histopathology with spatial transcriptomics. Nat. Methods.

[108] Xiang J., Wang X., Zhang X., Xi Y., Eweje F., Chen Y., Li Y., Bergstrom C., Gopaulchan M., Kim T. (2025). A vision-language foundation model for precision oncology. Nature.

[109] Xu Y., Wang Y., Zhou F., Ma J., Jin C., Yang S., Li J., Zhang Z., Zhao C., Zhou H. (2025). A multimodal knowledge-enhanced whole-slide pathology foundation model. Nat. Commun..

[110] Viswanathan V.S., Parmar V., Madabhushi A. (2024). Towards equitable AI in oncology. Nat. Rev. Clin. Oncol..

